# Proteomic Analysis of a Hypervirulent Mutant of the Insect-Pathogenic Fungus Metarhizium anisopliae Reveals Changes in Pathogenicity and Terpenoid Pathways

**DOI:** 10.1128/spectrum.00760-22

**Published:** 2022-10-31

**Authors:** Wenyou Huang, Peiquan Huang, Dan Yü, Chengzhou Li, Shuaishuai Huang, Ping Qi, Song Huang, Nemat O. Keyhani, Zhen Huang

**Affiliations:** a South China Agricultural University, College of Plant Protection, Key Laboratory of Bio-Pesticide Creation and Application of Guangdong Province, Guangzhou, China; b Department of Microbiology and Cell Science, Institute of Food and Agricultural Sciences, University of Florida, Gainesville, Florida, USA; c Guangzhou Institute for Food Inspection, Guangzhou, China; State Key Laboratory of Microbial Resources, Institute of Microbiology, Chinese Academy of Sciences

**Keywords:** proteomic analysis, UV mutant, *Metarhizium anisopliae*, terpenoid pathways

## Abstract

Metarhizium anisopliae is a commercialized entomopathogenic fungus widely used for the control of insect pests. Significant efforts have been expended to screen and/or select for isolates that display increased virulence toward target insect hosts. UV-induced mutagenesis has resulted in the isolation of a number of hypervirulent M. anisopliae mutants; however, the underlying mechanisms that have led to the desired phenotype have yet to be characterized. Here, we performed a comparative proteomic analysis of an M. anisopliae UV-induced hypervirulent mutant (MaUV-HV) and its wild-type parent using tandem mass tag (TMT)-based quantitative proteomics. A total of 842 differentially abundant proteins were identified, with 360 being more abundant in the hypervirulent mutant and 482 in the wild-type parent. In terms of differential abundance, the critical pathways affected included those involved in secondary metabolite production, virulence, and stress response. In addition, a number of genes involved in terpenoid biosynthesis pathways were identified as significantly mutated in the MaUV-HV strain. In particular, mutations in the farnesyl pyrophosphate synthase (*FPPS1*) and geranylgeranyl diphosphate synthase (*GGPPS5*) genes were seen. The effects of the *FPPS1* mutation were confirmed via the construction and characterization of a targeted gene knockout strain (Δ*MaFPPS1*). The overall effects of the mutations were increased resistance to UV stress, faster growth, and increased virulence. These results provide mechanistic insights and new avenues for modulating fungal virulence in efforts to increase the biological control potential of insect-pathogenic fungi.

**IMPORTANCE** The mechanisms that underlie and contribute to microbial (fungal) virulence are known to be varied; however, the identification of contributing pathways beyond known virulence factors remains difficult. Using TMT-based proteomic analyses, changes in the proteomes of an M. anisopliae hypervirulent mutant and its wild-type parent were determined. These data revealed alterations in pathogenicity, stress, and growth/developmental pathways, as well as pathways not previously known to affect virulence. These include terpenoid pathways that can be manipulated to increase the efficacy of fungal insect biological control agents for increased sustainable pest control.

## INTRODUCTION

Metarhizium anisopliae is a broad-host-range insect pathogen used as a biocontrol agent for the control of agriculturally important insect pests and has become a model system for studying emerging aspects of host-pathogen interactions (1–4). A number of M. anisopliae commercial products have been registered worldwide as environmentally friendly alternatives to chemical insecticides for pest management in a wide range of agricultural systems and beyond (5–8). However, the ability of entomopathogenic fungi to control insect pests in field applications is often hampered by various abiotic stresses, including the requirement by conidiospores of high relative humidity in order to germinate and the sensitivities of these cells to damage and loss of efficacy in response to UV irradiation and high temperatures (>32°C) (9–11). A number of different strategies have been employed to increase either stress resistance(s) and/or virulence, including genetic engineering, characterization of natural isolates, optimization of growth conditions of spores, and/or chemical/UV mutagenesis and screening (6, 9, 12, 13). Within the latter context, a number of studies have examined the effects of UV irradiation on M. anisopliae virulence (14–16), and the selection of mutants resistant to the nonmetabolizable glucose analog 2-deoxyglucose after UV irradiation resulted in mutants that displayed increased virulence (17).

In our previous study, an M. anisopliae UV-irradiated mutant (MaUV-HV, for UV-induced hypervirulent mutant) was obtained after UV exposure and subsequent screening for increased vegetative growth (14). MaUV-HV was subsequently shown to possess increased resistance to UV and thermal stress, and more importantly, increased virulence, compared to these characteristics in the parent strain (14). This hypervirulent mutant showed alterations in protein constituents and secondary metabolite production, including increased production of the insect toxin destruxin A; however, global proteomic changes that may have occurred in the mutant have thus far not been examined. Here, we further explore the mechanism(s) that underlie the observed hypervirulence seen in MaUV-HV via comparative proteomic analyses. Our data revealed significant rearrangements in the proteomic profile of MaUV-HV that coincided with increases in proteins that participate in secondary metabolite production, virulence, and stress response. Analysis of the differentially abundant proteins using the Pathogen-Host Interactions (PHI) database revealed enrichment of over 390 virulence-related proteins, including cytochrome P450 enzymes, LysM effectors, a polyketide synthase, and thioredoxin, among others. In terms of increased levels or loss of protein products, the production of two notable proteins, namely, farnesyl pyrophosphate synthase (encoded by *FPPS1*) and geranylgeranyl diphosphate synthase (encoded by *GGPPS5*) were seen to be affected. Loss of the latter gene in M. anisopliae (*MaGGPPS5*) has recently been shown to result in enhanced virulence (18), and here, we report on the construction and characterization of a Δ*MaFPPS1* mutant strain that recapitulated some but not all of the phenotypic aspects of the MaUV-HV strain. Together, these data provide a key confirmation of the mechanisms underlying the increased virulence seen in the MaUV-HV mutant.

## RESULTS

### Determination of the optimum culture conditions for protein expression and fungal metabolite production.

We have previously reported the isolation of a UV irradiation M. anisopliae mutant (here designated MaUV-HV) isolated after screening for faster vegetative growth that was shown to be significantly more virulent than the wild-type (WT) parent, while also displaying increased UV and thermal stress tolerances (14). Here, in order to probe the underlying mechanisms that contribute to the phenotypes described above for the MaUV-HV strain, a comparative proteomic analysis was performed. To determine the optimum parameters for proteomic analyses, a set of initial experiments were performed, beginning with secreted proteins. The secretome profiles of M. anisopliae wild type and MaUV-HV were examined over a time course (3 to 7 days) of growth in Czapek-Dox (CZ) broth (CZB). Total protein was extracted from separated (cell-free) culture supernatants and mycelia using trichloroacetic acid (TCA) and acetone as described in Materials and Methods. As expected, protein concentrations increased with incubation time (Table S1 in the supplemental material). SDS-PAGE analyses of the proteins derived from fungal-cell-free supernatants revealed significantly different patterns of protein bands between the mutant and the wild-type parent (Fig. S1A). Among the major bands seen in Coomassie-stained gels, a protein of ~35 to 36 kDa was prominent in both the wild-type and mutant secretomes at 3 to 5 days and gradually decreased by 6 to 7 days of growth, particularly in the mutant (Fig. S1A). The wild type howed another prominent band (~32 to 34 kDa) that was noticeably absent in the mutant. Conversely, the mutant showed a strong band of ~26 to 27 kDa throughout the time course of growth that was absent in the wild type. With respect to the mycelial extracts, as expected due to the presence of more diverse protein constituents, SDS-PAGE analyses indicated significant overlap between the wild type and the mutant, although a number of differences were noted (Fig. S1B).

As mentioned, MaUV-HV displays an increased thermal stress tolerance. In order to examine effects of thermal stress on the fungal proteome, the wild-type and mutant strains were grown in a range of temperatures (23 to 31°C) (Table S2). The overall patterns of the protein content as examined by SDS-PAGE indicated several differences in the cell-free supernatant extracts between the wild type and mutant over the temperature range examined (Fig. S1C); however, few obvious differences were observed in the mycelial extracts of the wild type and mutant over the temperature range tested (Fig. S1D). Therefore, in order to obtain higher resolution for delineating differences in the proteomes of the wild type and the MaUV-HV mutant, fungal cultures were grown for 5 days in CZB (25 ± 1°C) and the total proteome subsequently analyzed as indicated below.

### Comparative proteomic analysis of MaUV-HV and the wild-type M. anisopliae parent strain.

**FIG 1:**
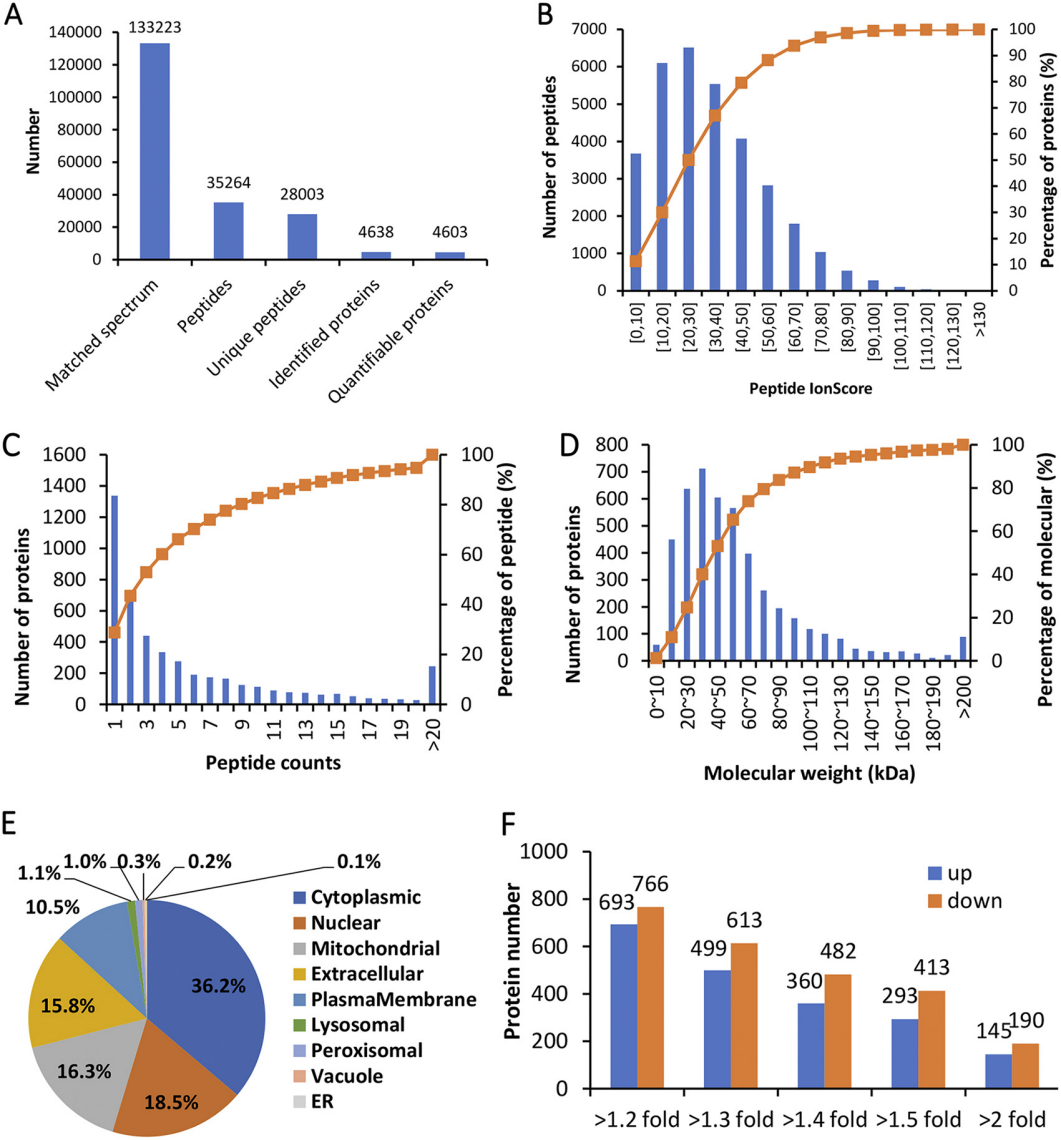
Statistical proteomics information from the TMT-based proteomics analysis. (A) Basic information statistics. (B) Peptide IonScore distribution. (C) Peptide count distribution. (D) Identified protein mass distribution. (E) Distribution of protein sequence coverage. (F) Numbers of differentially abundant proteins (DAPs) at different fold change values for up- and downregulated proteins.

The preliminary experiments detailed above indicated clear shifts in the proteome of the MaUV-HV strain compared to that of its wild-type parent. In order to identify these proteomic differences, the proteomes of the M. anisopliae wild type and MaUV-HV mutant (derived from mycelia grown for 5 days in CZB at 25 ± 1°C, and including both mycelial and secreted proteins in the sample analyses, mixed 1:1) were sequenced using tandem mass tag (TMT)-based quantitative proteomics. Totals of 35,264 peptides, 28,003 unique peptides, and 4,638 proteins were detected from the 133,223 matched spectra, and 4,603 identified peptides were quantified (Proteome Xchange identification no. [ID] PXD033657 or https://www.iprox.cn/page/project.html?id=IPX0004355000) (Fig. 1A and B). Most of the proteins were defined by more than two peptides, and the largest number of identified proteins were between 10 and 90 kDa in size (Fig. 1C and D). Approximately 87% of the proteins were identified with greater than 15% sequence coverage, with major categories including cytoplasmic, nuclear, mitochondrial, and extracellular proteins (Fig. 1E).

Comparative analyses indicated 842 proteins showing significant differences in protein abundance levels (ratios greater than 1.4 and less than 0.71 with a *P* value of <0.05), with 360 proteins more abundant in the MaUV-HV strain than in the wild type and 482 proteins less abundant in the mutant than in the parent strain (Fig. 1F). Three proteins (a glycosyl transferase and two uncharacterized proteins) were only detected in the MaUV-HV strain, and two proteins (Exs, related to the G protein signal transduction protein, and a BTB domain [zinc finger]/ankyrin repeat-containing protein) were only detected in the wild-type strain. Of the differentially abundant protein (DAP) data set of proteins that were more abundant in the MaUV-HV mutant, 6.28% (145/2,306 proteins) were increased 2-fold or less, 5.5% (127 proteins) between 2- and 4-fold, and 0.91% (21 proteins) >4 fold. Of the DAPs that were less abundant in the MaUV-HV mutant, 8.21% (190/2,313 proteins) were decreased 2-fold or less, 7.48% (173 proteins) between 2- and 4-fold, and 1.04% (24 proteins) >4-fold (Fig. 1F).

### Bioinformatic analysis of the DAPs.

**FIG 2:**
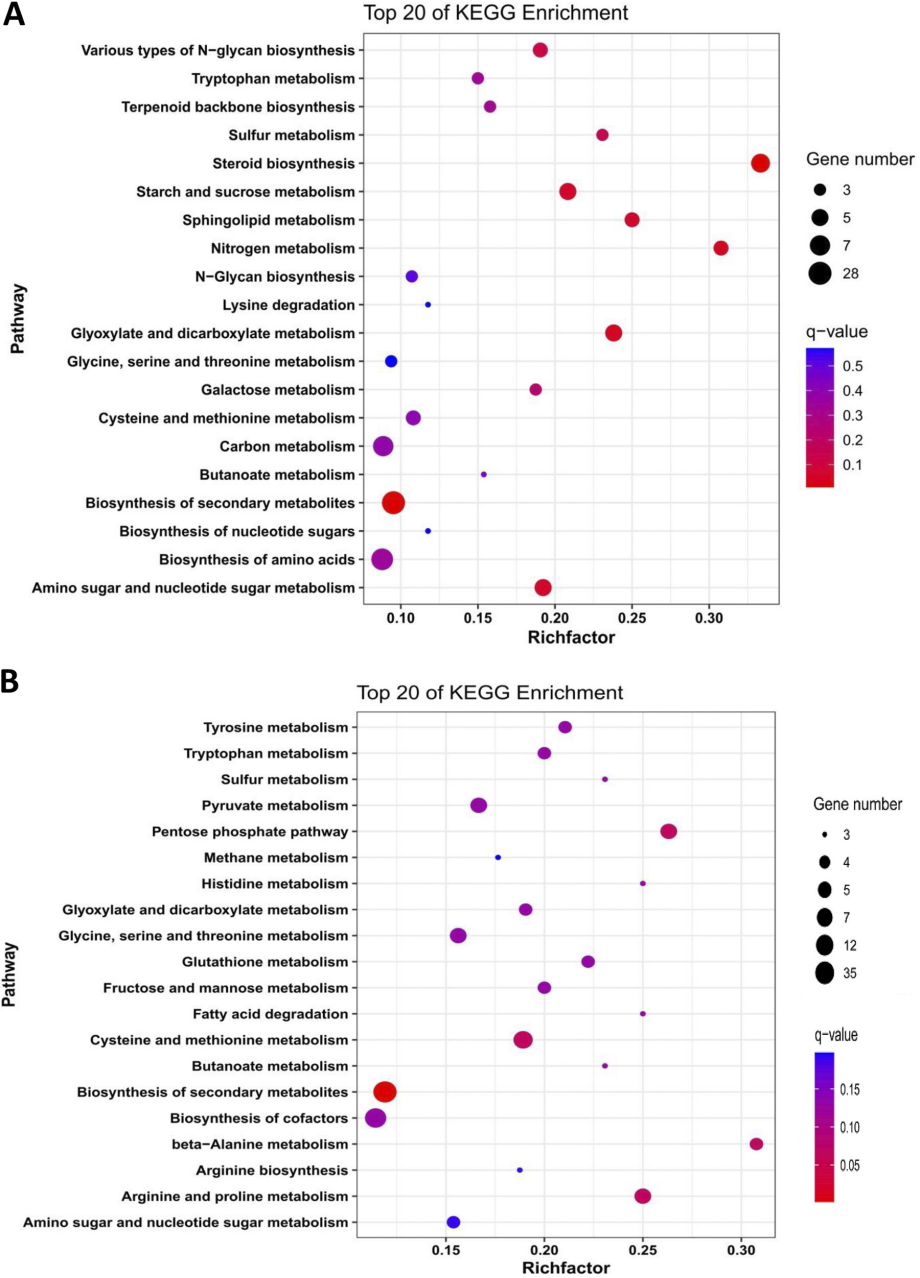
KEGG classification of differentially abundant proteins. The top 20 enriched categories are listed for upregulated proteins (A) and downregulated proteins (B).

**FIG 3:**
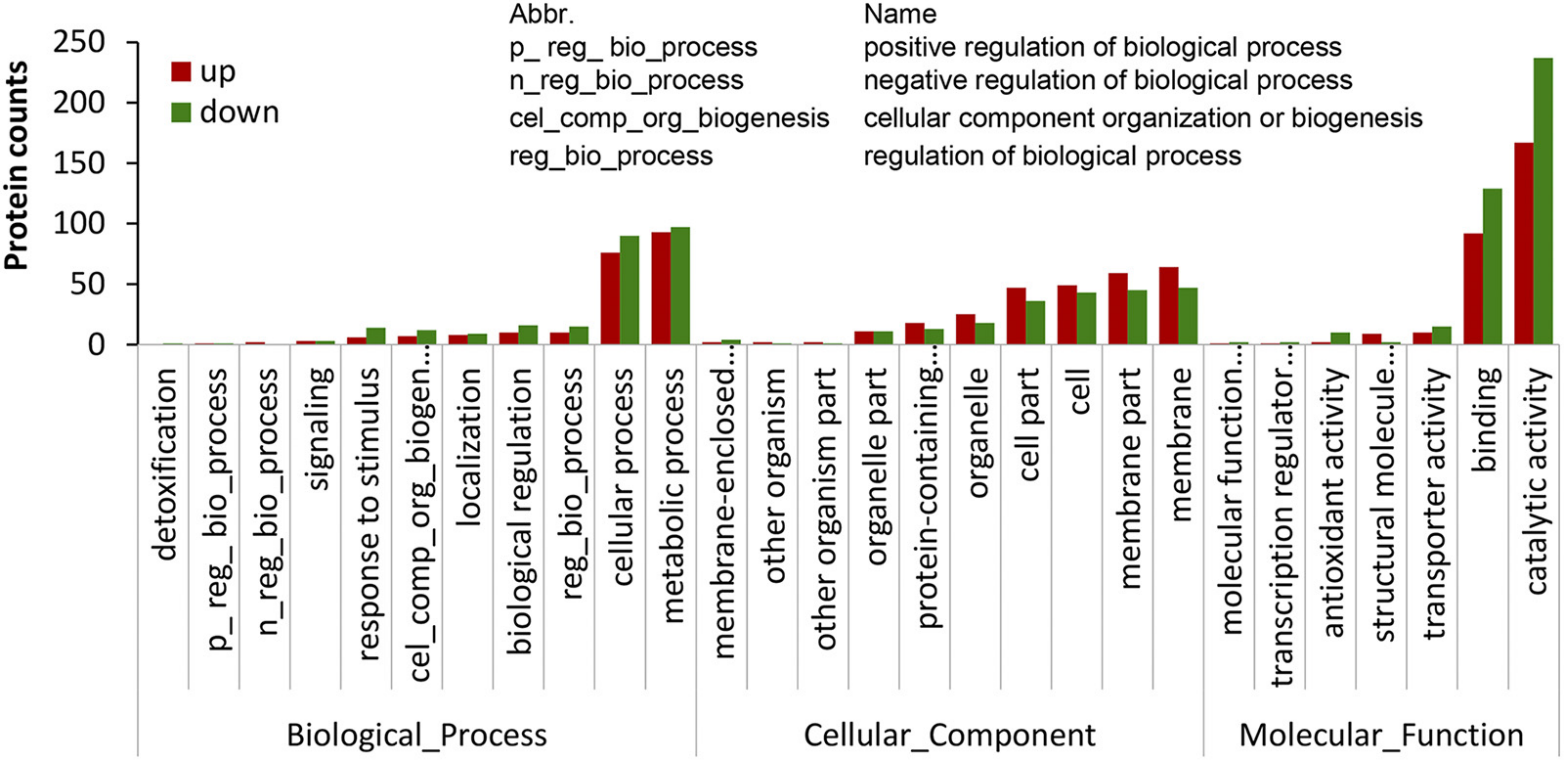
GO enrichment analysis in level 2 GO terms. Level 2 GO term enrichment is shown by bars, with upregulated DAPs in red and downregulated DAPs in green.

The set of 728 DAPs with a threshold of a log_2_(MaUV-HV/WT) of ≥0.5 or ≤−0.5 with a *P* value of <0.05 were annotated using Gene Ontology (GO) and Kyoto Encyclopedia of Genes and Genomes (KEGG) enrichment analyses. Of the annotated proteins, 313 were found to be more abundant in the MaUV-HV strain than in its wild-type parent, whereas 415 proteins were found to be less abundant in the MaUV-HV strain. With respect to the (313) more abundant DAPs found in the MaUV-HV mutant, the KEGG classifications indicated that those with the highest significance (lowest *q* value) were distributed within 5 broad categories, including biosynthesis of secondary metabolites, biosynthesis of amino acids, ribosome, carbon metabolism, and steroid biosynthesis (Fig. 2A). For the set (415) of less abundant DAPs found in the MaUV-HV mutant, the KEGG classifications indicated that those with the highest significance were distributed within 7 broad categories, including biosynthesis of secondary metabolites, biosynthesis of cofactors, cysteine and methionine metabolism, pentose phosphate pathway, arginine and proline metabolism, pyruvate metabolism, and glycine, serine and threonine metabolism (Fig. 2B). GO enrichment analyses showed similar profiles between the more and less abundant DAPs within the “Biological Processes” category, with the exception of “metabolic process and cellular process,” which had a greater distribution of less abundant DAPs than of more abundant ones (Fig. 3). In terms of “Molecular Function” categories, less abundant DAPs were found in greater numbers in the catalytic activity and binding categories, and also in transporter activity, antioxidant activity, molecular function regulator, and transcription regulation activity categories, whereas more abundant DAPs were in greater numbers in the structural molecule activity category. In contrast, the majority of “Cellular Component” categories showed higher distributions of more abundant DAPs than did the less abundant DAP data set. Further refinement of GO terms revealed enrichment of ribosome/ribonuclear components, amide and peptide biosynthesis, and cytoplasmic processes for the more abundant DAP data set and major enrichment within catalytic activity and minor enrichments within metabolic membrane (including mitochondrial) and metabolic/cofactor biosynthesis pathways in the less abundant DAP data set (Fig. S2).

**FIG 4:**
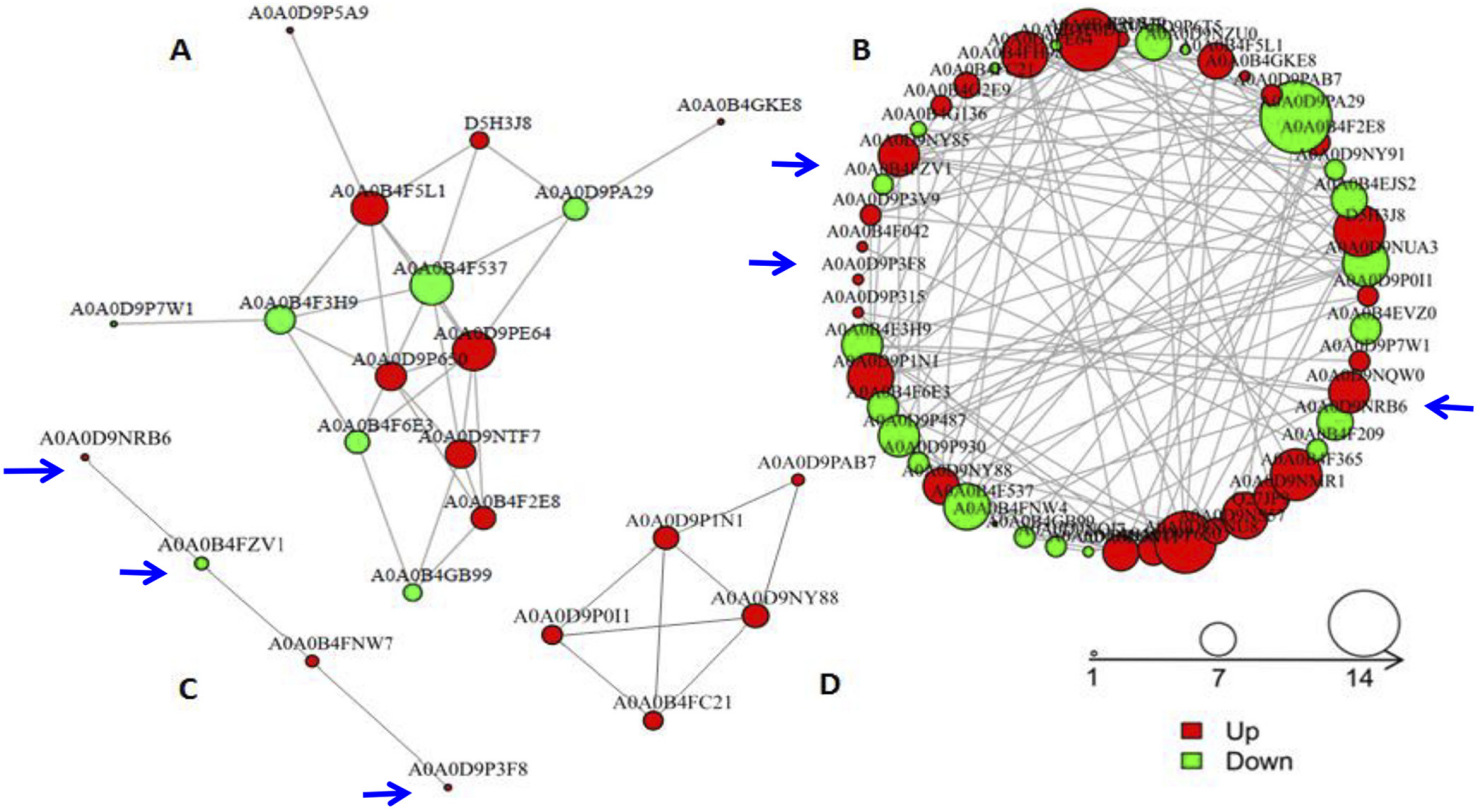
String network analysis of identified DAPs in MaUV-HV mutant strains. The green and red circles represent down- and upregulated DAP protein species, respectively. The size of the circle represents the number of DAPs within the protein-protein interaction. The top 100 proteins selected with the closest interaction relationships are mapped in the string network. The protein networks shown are biosynthesis of amino acids (A), biosynthesis of secondary metabolites (B), terpenoid backbone biosynthesis (C), and steroid biosynthesis (D).

To gain more information on the protein interaction networks between the members of the identified set of differentially abundant proteins, the 728 DAPs were analyzed using the String database. In order to clearly show protein-protein interaction relationships, the top 100 closest interactions were mapped within protein interaction networks (Fig. 4; proteins more abundant in the MaUV-HV mutant are in red, and those less abundant in the mutant are in green). Consistent with the overall DAP distributions, the protein interaction network identified relationships between proteins mainly involved in biosynthesis of amino acids (Fig. 4A; 14 proteins), secondary metabolites (Fig. 4B; the largest network identified, 63 proteins), and biosynthesis of cofactors, carbon metabolism, cysteine and methionine metabolism, and ribosome biosynthesis, as well as terpenoid (Fig. 4C; note that this sequence shows a linear interaction pathway involving four proteins) and steroid biosynthesis (Fig. 4D; a small five-protein network). Most of the proteins involved in biosynthesis of secondary metabolites, including geranylgeranyl diphosphate synthase (ID A0A0D9NRB6), metallopeptidase (A0A0D9P3F8), biotin carboxylase 3 (A0A0D9NUA3), hydroxymethylglutaryl coenzyme A synthase (A0A0B4FNW7), nudix hydrolase domain-containing protein (A0A0D9NMR1), and sulfate adenylyltransferase (A0A0D9NNU8), were more abundant in the hypervirulent mutant. Proteins related to biosynthesis of amino acids, including citrulline-aspartate ligase (A0A0B4F537), histidine biosynthesis trifunctional protein (A0A0B4F3H9), transketolase (A0A0D9PA29), ornithine transcarbamylase (A0A0B4F6E3), and phosphoserine aminotransferase (A0A0B4GB99), were less abundant in the mutant strain than in the wild-type parent.

### Protein expression profiles related to virulence.

**FIG 5:**
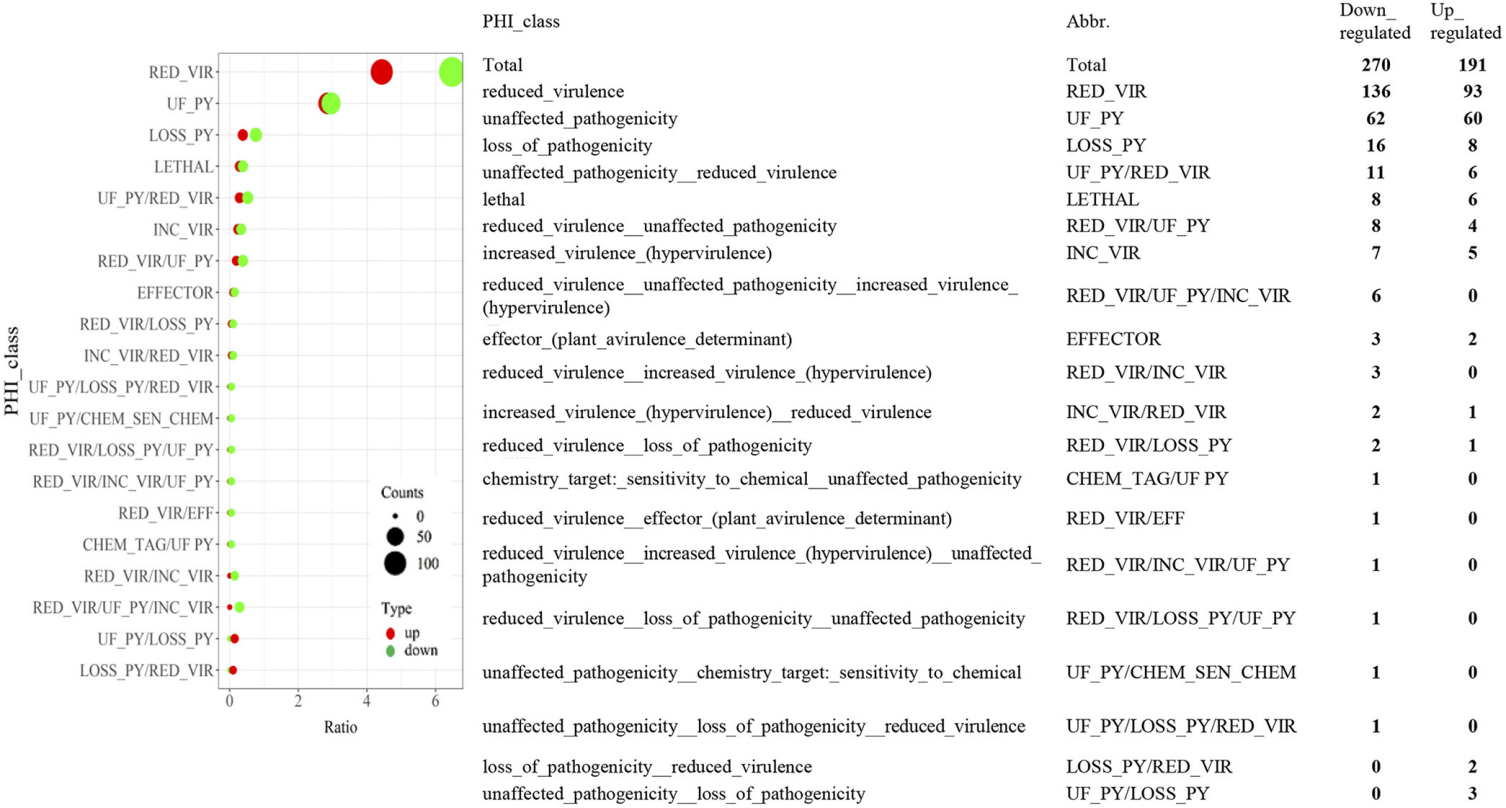
Pathogen-Host Interactions (PHI) database class and protein analysis of DAPs. PHI enrichment was performed with PHI-base version 4.10 as described previously. Up- and downregulated proteins were enriched into 7 main categories, with a few extra combined ones. Detailed PHI class and protein counts are listed, and abbreviations defined.

**FIG 6:**
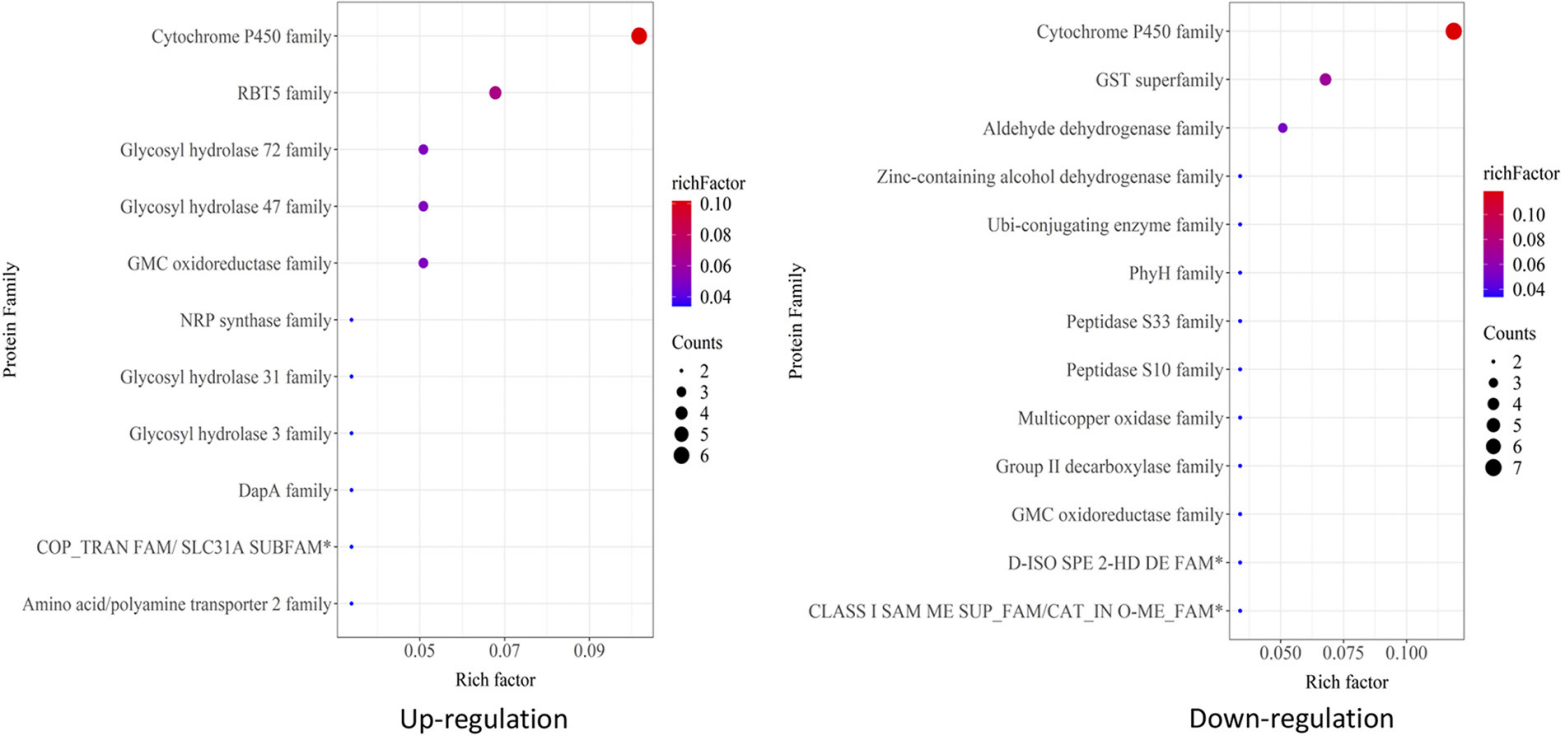
KEGG classification of differentially abundant protein families. The protein family analysis is shown; protein families with 2 or more members were selected for enrichment analysis. Gene set enrichment analysis (GSEA) was also performed.

**FIG 7:**
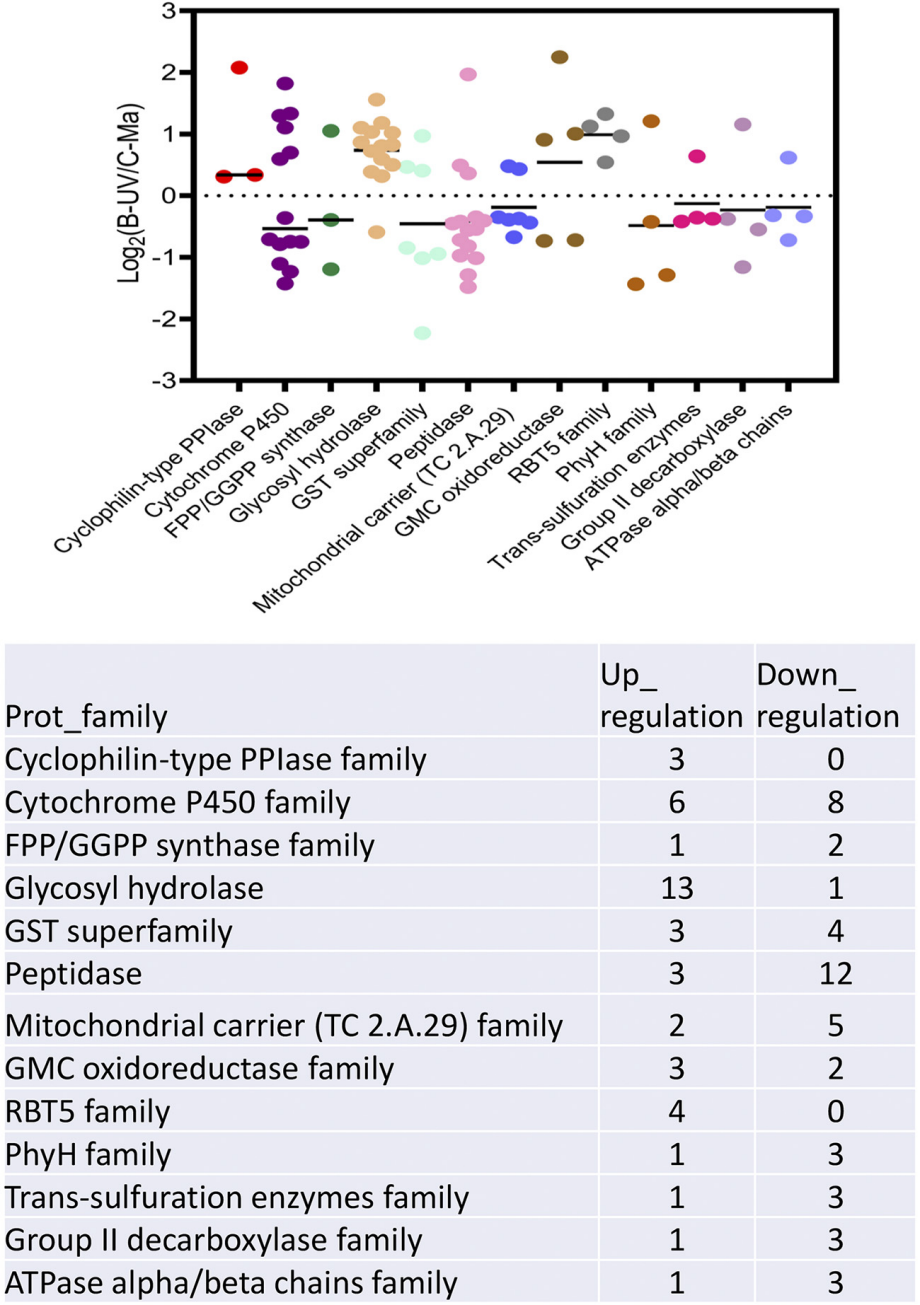
Overrepresented protein families were selected for further visualization of the expression pattern of differentially expressed proteins in proteomes. Protein family annotation was collected from UniProt database.

In order to probe proteomic changes related to virulence, the more and less abundant protein data sets were analyzed using the Pathogen-Host Interactions (PHI) database (Fig. 5). A total of 265 (36.4% of the total) less abundant and 183 (25.1% of the total) more abundant proteins could be annotated into one of the PHI categories. Overall, more and less abundant DAPs appeared roughly similarly distributed in the different virulence and pathogenicity processes. The majority of DAPs in either data set were distributed into reduced virulence and unaffected pathogenicity categories, although a number of effector, chemical sensitivity (exclusive to the less abundant DAP data set), and hypervirulence DAPs were also noted. Of lysosome-related proteins, two were found to be more abundant, FAD-dependent oxidoreductase (MAA_09919) and ribosomal protein S7e (MAA_07171), whereas aldehyde dehydrogenase (MAA_02517 and MAA_07506), alanine dehydrogenase (MAA_06003), acetyl-CoA acetyltransferase (MAA_06480), and saccharopine dehydrogenase-like protein (MAA_08309) were less abundant in the MaUV-HV strain than in the wild type. In addition, several pathogenicity factors, including cytochrome P450 family, RBT5 family, GMC oxidoreductase family, glycosyl hydrolase family, amino acid/polyamine transporter 2 family, copper transporter family, DapA family, and NRP synthase family, were also more abundant in the hypervirulent M. anisopliae mutant strain. Further analyses indicated less abundance of a different set of cytochrome P450 enzymes and a subset of glutathione *S*-transferase (GST) superfamily members, as well as dehydrogenase, GMC oxidoreductase, group II decarboxylase, multicopper oxidase, peptidase, PhyH, and ubiquitin-conjugating enzyme family members (Fig. 6). In addition, proteins that were less abundant in the MaUV-HV strain than in the wild-type parent included peptidases and two enzymes involved in terpenoid pathway biosynthesis, notably, a farnesyl pyrophosphate synthetase (FPPS) and a geranylgeranyl diphosphate synthase (GGPPS) (Fig. 7).

### Characterization of the nucleotide mutations of genes encoding select differentially abundant proteins implicated in terpenoid biosynthesis in MaUV-HV.

Since MaUV-HV was derived from a random (genomic) mutagenesis screen and preliminary data suggested altered secondary metabolite production in the mutant (14), we screened genes involved in the terpenoid pathway for mutations. In total, 11 DAPs were identified as being involved in the terpenoid biosynthesis pathway in the MaUV-HV mutant strain (Table 1). Among the 11 differentially expressed proteins, 5 were less abundant and 6 were more abundant in the MaUV-HV strain than in the wild type (Table 1). Of these, coding sequences from 9 genes were successfully amplified from both the mutant and wild-type strains and sequenced (Table 1; Table S3). Attempts to amplify the remaining two genes by PCR were unsuccessful. Alignments of sequences from the MaUV-HV and wild-type strains revealed a number of nucleotide changes that in several instances also resulted in the alteration of an open reading frame (ORF) and/or amino acids in the protein product (Table 1). Three genes, encoding hydroxymethylglutaryl coenzyme A synthase, acetyl-CoA acetyltransferase, and polyprenyl synthetase-related protein, showed no mutations in their sequences. The genes encoding six DAPs (four being more abundant and two less abundant in the MaUV-HV strain) showed mutations distributed along the sequence (Table 1). Of these, a mutation in the farnesyl pyrophosphate synthase gene (*FPPS1*, MAN_08993) indicated a change in the stop code (from TGA to GAT) that would be predicted to result in the further extension of the gene/protein from 1,032 bp/343 amino acids to 1,347 bp/448 amino acids. A mutation in the geranylgeranyl diphosphate synthase gene (*GGPPS5*, H634G_08383) resulted in changes in 5/331 amino acids (aa) (16/996 bp, Table 1). The highest number of mutations was in the prenylcysteine oxidase gene, which contained 25 different base pair changes (out of an ORF of 1,701 bp) that were predicted to result in 10 amino acid substitutions (out of a total of 566 aa).

**TABLE 1:** Summary of MaUV-HV mutations identified in terpenoid biosynthesis pathway

Protein name, ID (https://www.uniprot.org/)	Up- or downregulation, fold change	Gene designation, accession no.	Length of ORF/change in length (bp)	No. of aa/change in no. of aa; mutations
Farnesyl pyrophosphate synthetase, A0A0B4FZV1	Down, 0.4378	MAN_08993, XM_007826705.2	1,032/315^*a*^	343/112; Ile128→Thr128, Leu263→Ser263, Arg271→Gln271, Arg302→Gly302, Arg307→Gln307, Lys311→Arg311, Asp327→Gly327, Phe343→ASn343, 0→Asp344~Ser448^*b*^
Geranylgeranyl diphosphate synthase, A0A0D9NRB6	Up, 2.075	H634G_08383, XM_007825496.1	996/16	331/5; Thr3→Pro3, Ala7→Thr7, Asp44→Ala44, Thr62→Ala62, Glu304→Lys304
Hydroxymethylglutaryl-CoA reductase, A0A0B4GDP2	Up, 2.38	MAN_04675, XM_007818000.1	945/22	314/4; Tyr143→Phe143, Lys176→Arg176, Met206→Ile206, Val287→Leu287
Prenyltransferase, A0A0B4FY87	Up, 1.27	MAN_09004, XM_007824141.1	1,536/10	511/6; Lys129→Gln129, K145→Asn145, Ile198→Val198, Phe397→Ile397, Thr400→Ser400, Ile481→Val481
metallopeptidase, A0A0D9P3F8	Up, 2.78	H634G_04861, XM_007824163.1	1,371/10	456/2; Ala235→Thr235, Ser243→Ala243
Prenylcysteine oxidase, A0A0B4EYR9	Down, 0.773	MAN_09560, XM_007822770.1	1,701/25	566/10; Gly9→Val9, Gln20→Arg20, Arg35→His35, Ile47→Val47, Ala56→Glu56, Arg151→Lys151, Ala295→Thr295, Leu420→Phe420, Gln455→Glu455, Asp564→His564
hydroxymethylglutaryl coenzyme A synthase, A0A0B4FNW7	Up, 1.41	MAN_04159, XM_007820017.1	1,362/0	453/0
acetyl-CoA acetyltransferase, A0A0B4FTF6	Down, 0.792	MAN_00586, XM_007824478.1	1,293/0	430/0
Polyprenyl synthetase-related protein, A0A0D9NQA4	Down, 0.7623	H634G_08714, XM_007821018.1	1,294/0	429/0
Phosphomevalonate kinase, A0A0D9P644	Up, 1.27	H634G_04534		No PCR product obtained
STE24 endopeptidase, A0A0B4F5W9	Down, 0.5079	MAN_09026		No PCR product obtained

a Mutations result in extension of ORF to 1,347 bp.

b Mutations result in a total of 448 amino acids.

### The Δ*MaFPPS1* mutant strain displays faster germination, faster vegetative growth, and increased conidial resistance to UV stress.

The proteomic data set coupled to DNA sequence analysis of specific loci implicated mutations in a farnesyl pyrophosphate synthase (annotated as *MaFPPS1*, A0A0B4FZV1) and a geranylgeranyl diphosphate synthase (*MaGGPPS5*) that putatively could contribute to the phenotype of the MaUV-HV strain. The *MaGGPPS5* gene has recently been characterized (18), and we therefore sought to test any contributions of *MaFPPS1* in mediating phenotype(s) of the MaUV-HV strain. In order to do so, a targeted gene knockout (Δ*MaFPPS1*) mutant, a complemented (Δ*MaFPPS1*::*MaFPPS1*) mutant, and a constitutive expression mutant (*MaFPPS1*^Const^, with the *MaFPPS1* ORF under the control of the *TrpC* promoter) were constructed in the wild-type strain as detailed in Materials and Methods. An initial PCR screen followed by Southern blot analyses were used to confirm single integration events in the transformed strains, and the *MaFPPS1* expression levels in the different strains were confirmed by quantitative real-time (RT)-PCR analyses (Fig. S3). The latter data showed complete loss of *Ma*FPPS1 expression in the Δ*MaFPPS1* strain and an ~40 to 60% increase in *Ma*FPPS1 expression in the *MaFPPS1*^Const^ strain.

**FIG 8:**
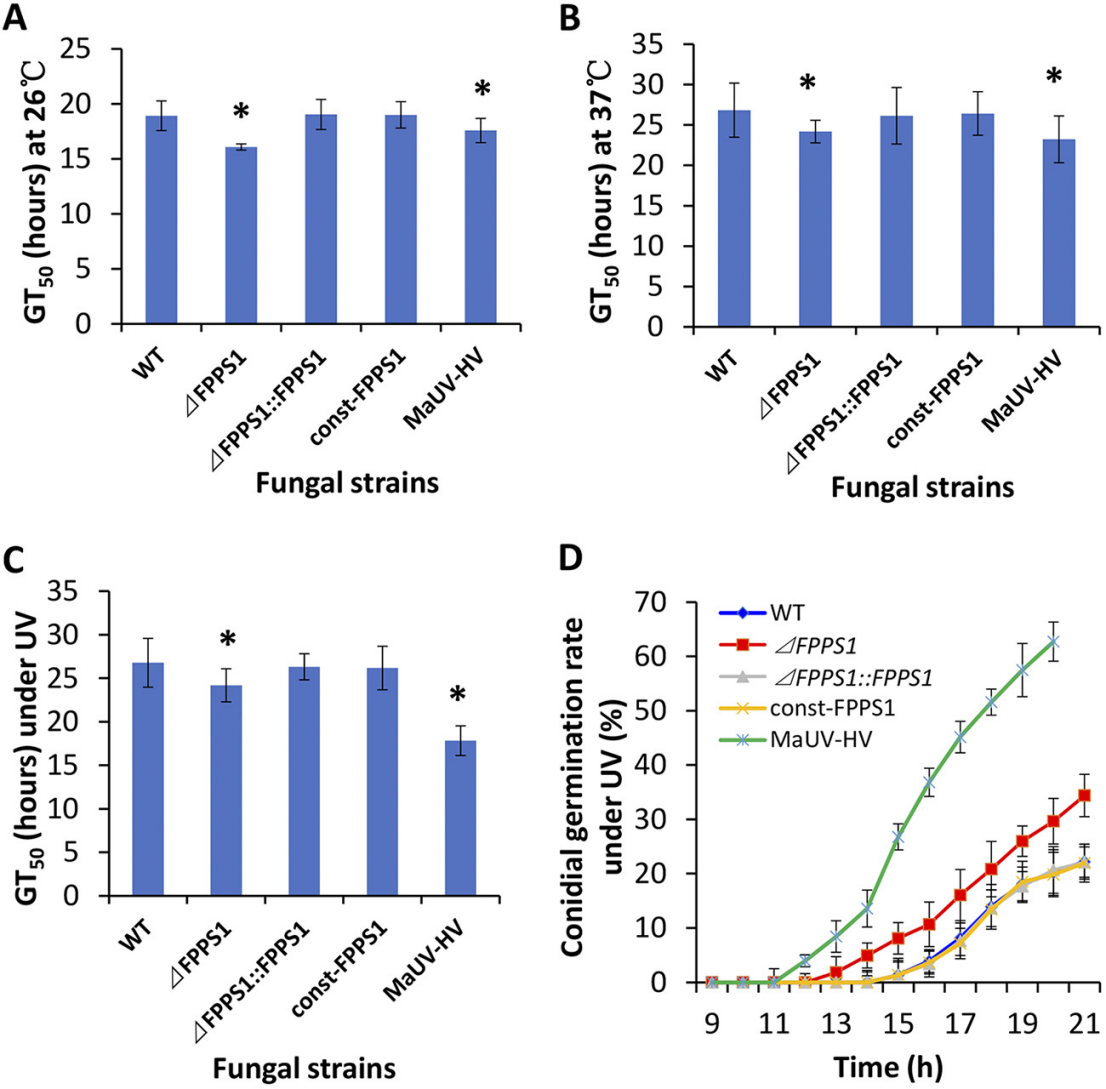
Phenotypic characterization of the responses of the different M. anisopliae strains to heat and UV stress. (A) GT_50_ assay of M. anisopliae strains, including the WT and *ΔFPPS1*, *ΔFPPS1*::*FPPS1*, const-*FPPS1* (*MaFPPS1* constitutive expression strain [*MaFPPS1*^Const^ in the text]), and MaUV-HV mutants, cultured at 26°C without UV irradiation. GT_50_, time for 50% germination of conidia in CZB. (B) Bioassay of different strains under heat stress. (C) Bioassay of different stains under UV-B irradiation stress. (D) Conidial germination of different strains in CZB at 26°C under UV. Experiments were repeated three times. Error bars show ±SE. *, significant difference at *P* < 0.05.

**FIG 9:**
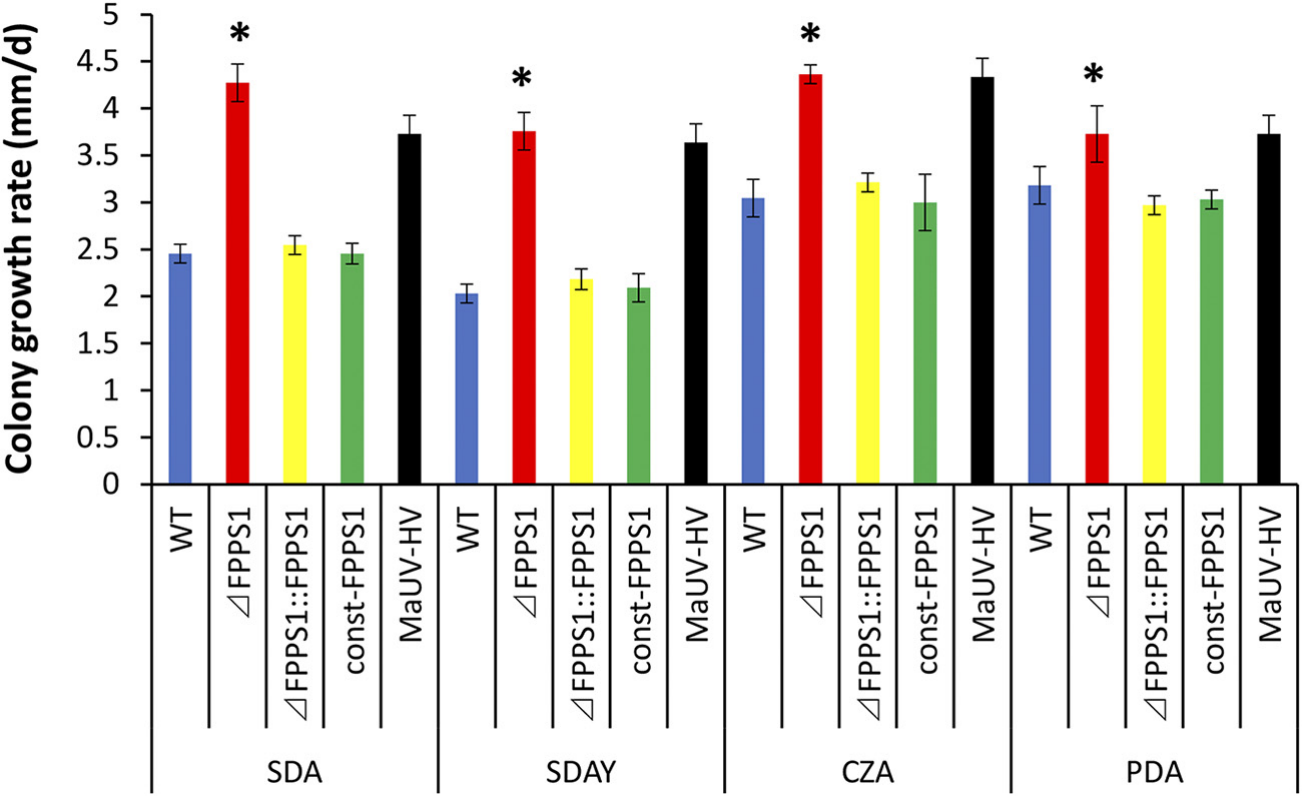
Comparing the colony growth rates among different M. anisopliae strains on different media. Different fungal strains, including the WT and *ΔFPPS1*, *ΔFPPS1*::*FPPS1*, const-*FPPS1* (*FPPS1* constitutive expression strain), and MaUV-HV mutants, were inoculated onto PDA, CZA, SDAY, and SDA media and incubated for 12 days at 26°C. All experiments were repeated three times. Error bars indicate ±SE. *, significant difference at *P* < 0.05.

Under three different conditions tested (using standard medium for all), including growth (i) at 26°C (standard temperature), (ii) after heat shock (HS) (37°C), and (iii) after exposure to UV irradiation for 30 min, as detailed in Materials and Methods, conidia derived from the Δ*MaFPPS1* strain germinated significantly (*P* < 0.01) earlier and to a greater extent than those of the wild-type or complemented strains over the time course measured. When grown at 26°C (without UV irradiation), the mean times for 50% germination (GT_50_) of conidia from the Δ*MaFPPS1* (and MaUV-HV) strains in Czapek-Dox broth (CZB) with 0.5% peptone (CZP) were significantly shorter (15 to 20%) than those of the parent wild type and complemented strain (Fig. 8A). A similar but slightly more moderate effect (10 to 15% reduction in GT_50_; *P* < 0.5) was seen for the Δ*MaFPPS1* mutant cultured at 37°C compared to controls (Fig. 8B). After UV exposure, germination was visible within 13 h postinoculation into CZP for the Δ*MaFPPS1* strain, whereas similar germination levels were not seen until >15 h for the control wild-type strain (*P* < 0.01) (Fig. 8D). However, MaUV-HV conidia remained more robust than those of either the Δ*MaFPPS1* or wild-type strain, with germination visible as early as 12 h postinoculation into medium. The calculated GT_50_ values in CZP after UV irradiation exposure of conidia were found to be 24.19 ± 1.9 h (mean ± standard error [SE]) for the Δ*MaFPPS1* mutant, compared to 26.8 ± 3.1 h and 26.3 ± 1.5 h for the parental wild type and complemented strain, respectively (Fig. 8C). The Δ*MaFPPS1* mutant strain also displayed faster vegetative growth at 26°C (*P* < 0.01) on all culture media tested, including Sabouraud dextrose agar (SDA), Sabouraud dextrose agar plus 0.5% yeast extract (SDAY), potato dextrose agar (PDA), and Czapek-Dox agar (CZA) (Fig. 9), compared to the control wild-type and complemented strains, and intriguingly, the Δ*MaFPPS1* strain grew faster than the MaUV-HV strain on SDA. However, constitutive expression of *MaFPPS1* did not affect vegetative growth in any of the media tested. Consistent with the faster mycelial growth seen on agar plates, the dry mycelial yield from fungal cultures grown in SDAY showed an ~15.2% (*P* < 0.01) increase in cell biomass, from 22.9 g/L and 23.3 g/L for the wild-type parent and complemented strains to 26.3 g/L for the Δ*MaFPPS1* mutant.

### The Δ*MaFPPS1* strain shows increased virulence.

Insect bioassays were performed using a variety of hosts and two different inoculation protocols, namely, (i) topical bioassays representing the “natural” route of infection and (ii) intrahemocoel injection assays that bypass the host cuticle to directly challenge the host immune system. For topical bioassays, nymphs and adults of the white-backed plant hopper (WBPH; Sogatella furcifera Horváth) were used. Mortality assays examining a time course of infection against WBPH adults treated with fungal conidia (1 × 10^7^ conidia/mL) indicated a sharp decrease (~44.6%, *P* < 0.01) in the lethal time to kill 50% of infected insects (LT_50_), from ~5.66 ± 0.43 days and ~5.91 ± 0.46 days for the complemented and wild-type strains, respectively, to 3.27 ± 0.21 days for the Δ*MaFPPS1* mutant (Table 2). These bioassays also indicated that the *MaFPPS1* constitutive expression mutant strain was modestly less virulent (~14.4%, LT_50_ = 6.69 ± 0.61 days) than the parental wild-type strain. In addition, compared to the LT_50_ for the MaUV-HV strain, the LT_50_ for the Δ*MaFPPS1* strain was ~27% lower (MaUV-HV LT_50_ = 4.5 ± 0.30 days). Calculations of the lethal dose for 50% mortality (LD_50_) using adult WBPH as the host and determined at 5- and 10-day-postinfection time points indicated that significantly lower conidial concentrations (indicating higher virulence) were needed for the Δ*MaFPPS1* mutant (~106.3- and 10-fold lower at 5 and 10 days, respectively; *P* < 0.001) than for the parental wild type, with LC_50_ values of ~5.85 × 10^5^ and 2.25 × 10^4^ conidia/mL at the 5- and 10-day postinoculation time points for the Δ*MaFPPS1* mutant and ~6.22 × 10^7^ and 2.24 × 10^5^ conidia/mL for the wild type at the same time points (Table 2). For the *MaFPPS1* constitutive expression mutant strain, the LC_50_ value was calculated to be 6.12 × 10^5^ conidia/mL (10 days postinfection), which was ~185% higher than that of the wild type. The calculated LC_50_ value for the original MaUV-HV isolate was 3.56 × 10^4^ conidia/mL (10 days postinfection), which was ~36.8% higher than that for the Δ*MaFPPS1* strain. Bioassays using WBPH nymphs showed similar (to adults) mortality trends with respect to the different fungal strains tested. Overall, the LC_50_ values for the 5- and 10-day-postinfection time points were significantly lower (i.e., higher virulence) for the Δ*MaFPPS1* mutant (~26.5- and 15.7-fold lower; *P* < 0.001) (Table 2) but ~205.3% higher (decreased virulence) for the *MaFPPS1* constitutive expression strain (2.00 × 10^6^ conidia/mL; *P* < 0.01) compared to the LC_50_ values for wild-type M. anisopliae. In terms of LT_50_ values, treatment of insect hosts with 1.0 × 10^7^ conidia/mL resulted in LT_50_ values of ~6.22 ± 0.54 days for the parental wild type and a modestly higher ~7.18 ± 0.65 days for the constitutive expression mutant strain. However, the LT_50_ value was sharply reduced, to 3.7 ± 0.24 days (~40.5% decrease), for the Δ*MaFPPS1* mutant strain (*P* < 0.001) (Table 2).

**TABLE 2:** Insect bioassays were performed to determine lethal concentration and lethal time for 50% mortality of target hosts

Insect stage	Fungal strain	Time (days)	Mean value ± SE (range) for:	
			LC_50_ (conidia/mL)	LT_50_ (days)
Adult	WT	5	6.22 × 10^7^ ± 8.33 × 10^7^ (4.53 × 10^6^, 8.56 × 10^8^)	5.91 ± 0.46 (5.07, 6.89)
		10	2.24 × 10^5^ ± 1.18 × 10^5^ (7.96 × 10^4^, 6.32 × 10^5^)	
	*ΔFPPS1*	5	5.85 × 10^5^ ± 2.60 × 10^5^ (2.45 × 10^5^, 1.40 × 10^6^)	3.27 ± 0.21 (2.88, 3.71)
		10	2.25 × 10^4^ ± 0.94 × 10^4^ (0.99 × 10^4^, 5.10 × 10^4^)	
	*ΔFPPS1*::*FPPS1*	5	3.35 × 10^7^ ± 3.6 × 10^7^ (4.05 × 10^6^, 2.76 × 10^8^)	5.66 ± 0.43 (4.87, 6.58)
		10	2.74 × 10^5^ ± 1.39 × 10^5^ (1.01 × 10^5^, 7.43 × 10^5^)	
	Const-*FPPS1*^*a*^	5	2.09 × 10^8^ ± 3.32 × 10^8^ (9.25 × 10^6^, 4.71 × 10^9^)	6.69 ± 0.61 (5.59, 8.0)
		10	6.12 × 10^5^ ± 3.60 × 10^5^ (1.93 × 10^5^, 1.94 × 10^6^)	
	MaUV-HV	5	3.28 × 10^6^ ± 3.28 × 10^6^ (4.63 × 10^5^, 2.32 × 10^7^)	4.5 ± 0.30 (3.94, 5.13)
		10	3.56 × 10^4^ ± 1.67 × 10^4^ (1.42 × 10^4^, 8.95 × 10^4^)	
4th instar	WT	5	2.17 × 10^7^ ± 2.09 × 10^7^ (3.27 × 10^6^, 1.43 × 10^8^)	6.22 ± 0.54 (5.25, 7.36)
		10	6.55 × 10^5^ ± 4.24 × 10^5^ (1.85 × 10^5^, 2.33 × 10^6^)	
	*ΔFPPS1*	5	8.19 × 10^5^ ± 4.80 × 10^5^ (2.59 × 10^5^, 2.58 × 10^6^)	3.7 ± 0.24 (3.26, 4.20)
		10	4.16 × 10^4^ ± 1.88 × 10^4^ (1.71 × 10^4^, 1.01 × 10^5^)	
	Const-*FPPS1*	5	2.78 × 10^7^ ± 2.42 × 10^7^ (5.10 × 10^6^, 1.52 × 10^8^)	7.18 ± 0.76 (5.83, 8.84)
		10	2.00 × 10^6^ ± 1.53 × 10^6^ (4.48 × 10^5^, 8.93 × 10^6^)	
	MaUV-HV	5	4.03 × 10^6^ ± 3.54 × 10^6^ (7.24 × 10^5^, 2.25 × 10^7^)	4.95 ± 0.32 (4.35, 5.59)
		10	7.58 × 10^4^ ± 3.38 × 10^4^ (3.15 × 10^4^, 1.82 × 10^5^)	

a The const-*FPPS1* strain is the *MaFPPS1* constitutive expression strain (*MaFPPS1*^Const^ in the text).

**FIG 10:**
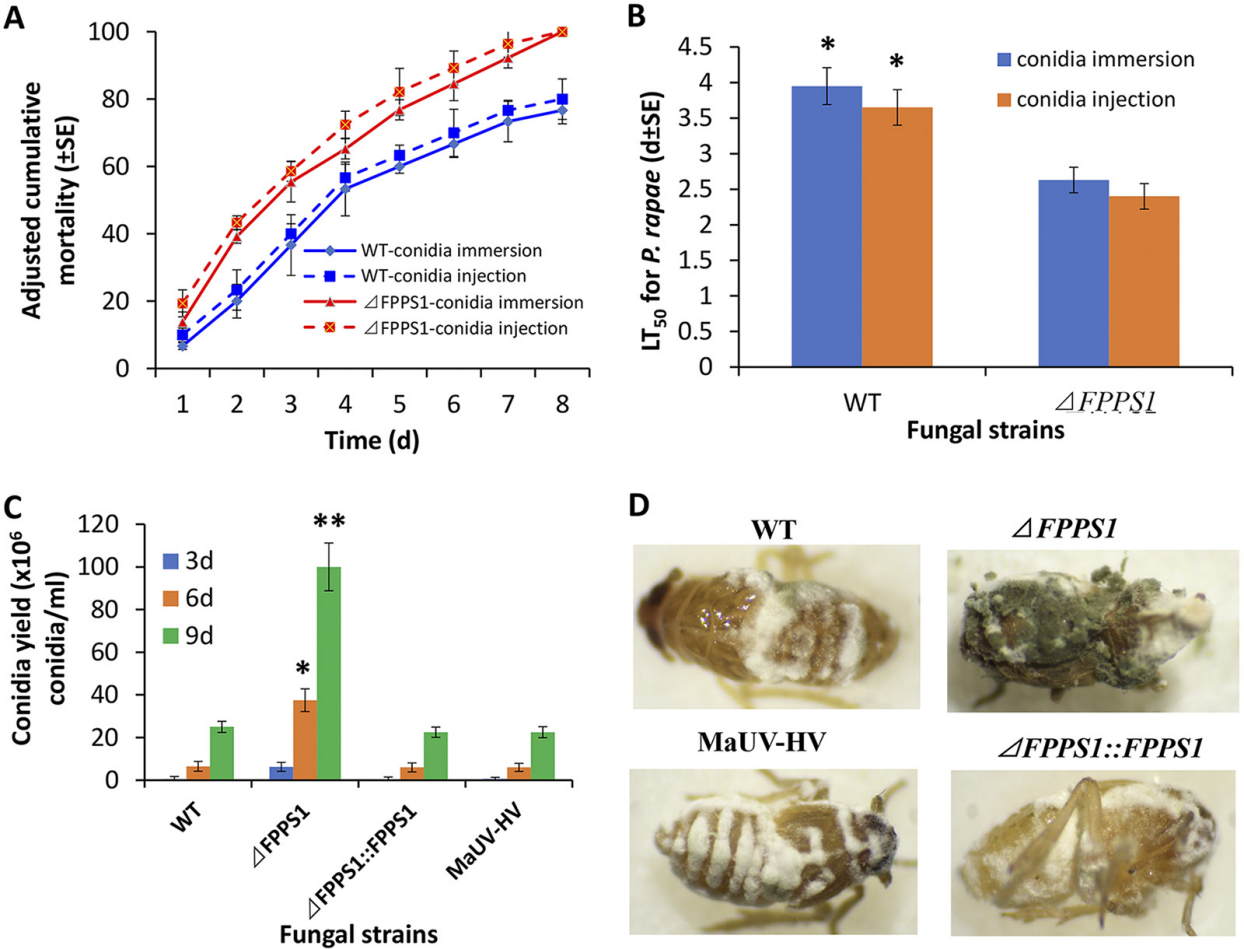
Insect bioassays and fungal sporulation on cadavers. (A) Time course of P. rapae larva mortality after topical infection with a conidial suspension (1 × 10^7^ conidia/mL) of M. anisopliae wild-type strain or the *ΔFPPS1* mutant or intrahemocoel injection of conidial suspensions (1 × 10^7^ conidia/mL,10 μL/larvae) of the same strains. (B) LT50 values of conidial suspensions from *ΔFPPS1* and wild-type strains against P. rapae larvae. (C) Calculated conidial production on insect cadavers at 3, 6, and 9 days postinfection of 4th instar nymphs of S. furcifera treated with different fungal strains and maintained at 26°C and in relative humidity of >90%. (D) Images of insect cadavers of S. furcifera killed by different strains. Experiments were performed in triplicate. Error bars indicate ±SE. *, *P* < 0.05; **, *P* < 0.01.

Larvae of the cabbageworm, Pieris rapae, were also used as target hosts, using both topical and intrahemocoel injection methods. Experiments were performed examining a time course from 1 to 8 days postinoculation for treatments using 10^7^ conidial/mL for topical bioassays and 1 × 10^5^ conidia/insect for intrahemocoel injection bioassays. Calculated adjusted mortality analyses indicated that the Δ*MaFPPS1* strain showed increased mortality for both injection and topical treatments, with LT_50_ values of 4.0 ± 0.25 days (topical) and 3.7 ± 0.25 days (injection) for the wild type and 2.7 ± 0.18 days (topical) and 2.4 ± 0.18 days (injection) for the Δ*MaFPPS1* mutant strain (*P* < 0.001, ~34% decrease in LT_50_) (Fig. 10A and B).

Visual inspection of host cadavers revealed more vigorous mycelial fungal growth and greater levels of green conidial pigmentation (reflecting conidial production) on insects inoculated with the Δ*MaFPPS1* mutant than on insects inoculated with the wild-type M. anisopliae or complemented strains (Fig. 10D). Quantification of the production of conidia on insect cadavers killed by fungal infection revealed significantly earlier conidiation onset (i.e., occurring at 3 days postmortality for the Δ*MaFPPS1* strain) and an ~4- to 6-fold-greater conidial yield on host cadavers measured at 6 and 9 days postmorbidity from infections due to the Δ*MaFPPS1* mutant than from infections due to the wild-type and complemented strains (*P* < 0.001) (Fig. 10C).

### The Δ*MaFPPS1* strain accumulates subglutinol and destruxin insect toxins.

Organically (ethyl acetate) derived extracts of the wild-type (WT), MaUV-HV, and Δ*MaFPPS1* strains grown in CZB supplemented with 0.5% peptone (CZP) were prepared for analyses of secondary metabolites as detailed in Materials and Methods. High-performance liquid chromatography (HPLC) and LC/mass spectrometry (MS) analyses were used to identify compounds (8 in total) related to subglutinols and destruxins (structures are shown in Fig. S4). These included destruxins A, A2, dihydro-A, B, and B2 (note that, based on the method used, the B2 and dihydro-A enantiomers could not be separated), subglutinols C and analog 45, and an uncharacterized compound. Subglutinols C and analog 45 were detected in wild-type extracts; however, the latter compound was not detected in Δ*MaFPPS1* extracts (Table 3). Extracts from the Δ*MaFPPS1* mutant contained ~10-fold-higher levels of destruxins D, B, and A, ~17.5-fold-higher levels of destruxin A2, and ~47.7-fold-higher levels of destruxin B2/dihydro-A than extracts derived from the WT strain (*P* < 0.001) (Table 3). The measured levels of destruxins A, A2, B, B2/dihydro-A, and D were similar between the Δ*MaFPPS1* mutant and the MaUV-HV strain. In addition, a 110-fold increase in the uncharacterized metabolite was seen for the former strain.

**TABLE 3:** Metabolites identified in cell-free extracts from different fungal strains

Compound	Retention times (min) for UV*/*MS spectra	*m*/*z*	*M. anisopliae* strain:		
			Δ*FPPS1* strain(cps)^*a*^	WT (cps)	MaUV-HV (cps)
Subglutinols					
C	4.54/4.75	443.2792	2.17 × 10^7^	No detection	1.97 × 10^7^
Analog 45	8.31/8.38	365.2839	No detection	3.77 × 10^6^	No detection
Destruxins					
A	4.57/4.69	578.36542	7.09 × 10^9^	7.33 × 10^8^	8.03 × 10^9^
B	5.46/5.66	594.39687	6.38 × 10^9^	5.29 × 10^8^	5.93 × 10^9^
D	4.23/4.36	624.37177	3.20 × 10^9^	2.99 × 10^8^	3.29 × 10^9^
A2	4.23/4.33	564.35016	2.35 × 10^9^	1.34 × 10^8^	2.01 × 10^9^
B2 and dihydro-A^*b*^	5.12/5.18	580.38168	6.91 × 10^9^	1.45 × 10^8^	7.85 × 10^9^
Unidentified	7.32/7.39	509.29932	2.24 × 10^9^	2.03 × 10^7^	8.07 × 10^8^

a cps means the peak high of MS.

b Destruxins B2 and dihydro-A are enantiomers that are not distinguished by our detection methods.

**FIG 11:**
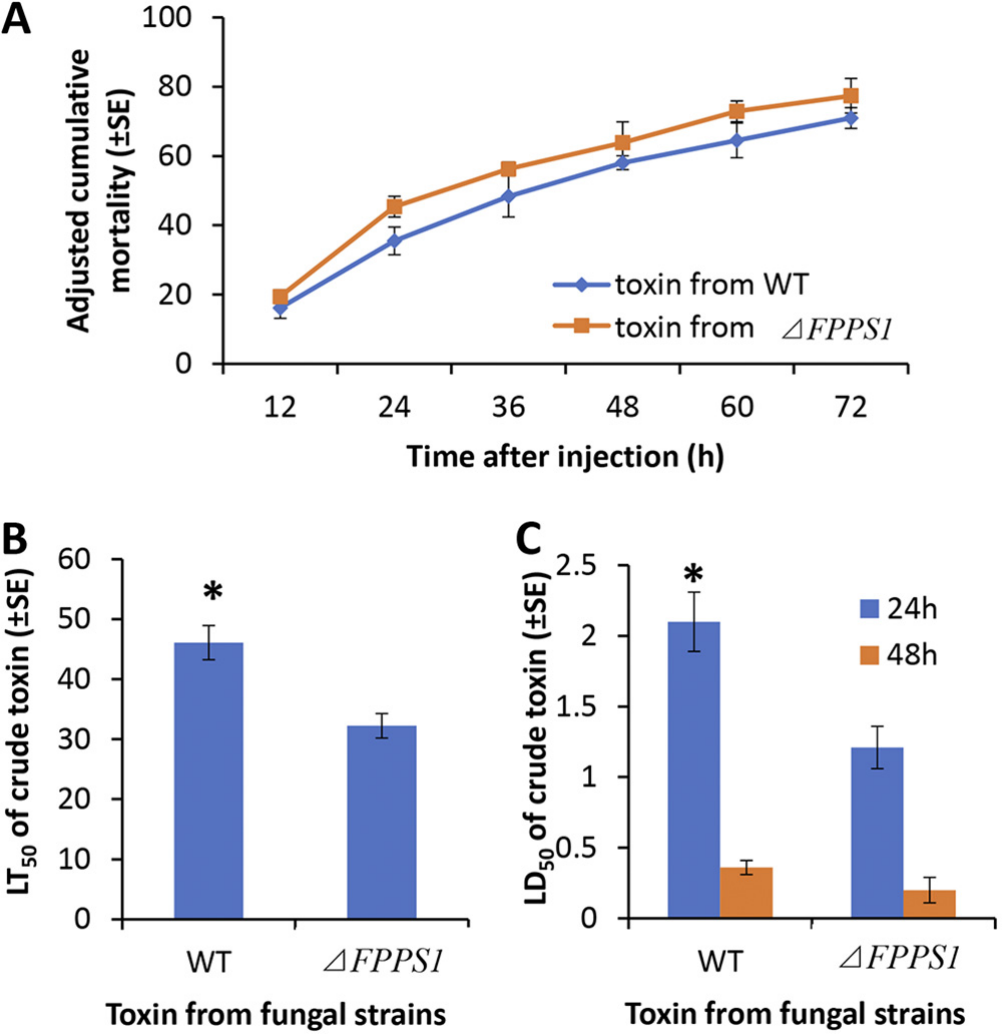
Injection (intrahemocoel infection) bioassays using insect host P. rapae were performed to test the toxicity of the cell-free extracts as detailed in Materials and Methods, examining the time course (12 to 72 h) of mortality after treatment. (A) Time course of P. rapae larva mortality after intrahemocoel injection with crude toxin (10 μL, 0.5 ppm) derived from Δ*FPPS1* and wild-type strains. (B) LT_50_ values of injected (10 μL, 0.5 ppm) toxin extracts from *ΔFPPS1* and wild-type strains. (C) LD50 values of injected crude toxin extracts from *ΔFPPS1* and wild-type strains. Experiments were performed in triplicate. Error bars indicate ±SE. *, *P* < 0.05.

Injection (intrahemocoel infection) bioassays using the insect host P. rapae were performed to test the toxicity of the cell-free extracts as detailed in Materials and Methods, examining the time course (12 to 72 h) of mortality after treatment. These injection assays indicated significantly (*P* < 0.001) increased toxicity of the mutant extracts compared to that of the wild-type extracts over the entire time course of the experiment. The calculated LT_50_ values for the different cell-free extracts (used at 0.5 ppm) were 46.1 ± 2.9 h for the wild-type strain, but they were significantly lower, 32.2 ± 2.0 h, for the Δ*MaFPPS1* mutant (indicating an ~30% increase in toxicity; *P* < 0.001) (Fig. 11A and B). Experiments performed to compare mean lethal dose concentrations revealed the calculated LD_50_ values of the extracts toward P. rapae larvae determined at 24 and 48 h postinjection to be 2.1 ± 0.2 and 0.36 ± 0.05 ppm for the wild-type parental strain and 1.21 ± 0.15 and 0.20 ± 0.09 ppm for extracts derived from the Δ*MaFPPS1* strain (representing an ~44% increase in insecticidal toxicity) (Fig. 11C).

## DISCUSSION

Although entomopathogenic fungi are viable alternatives to chemical pesticides for pest control, low tolerances to abiotic stress and the length of time it takes for the pathogen to kill hosts (3- to 7-day period) can limit the application of these organisms in many settings. The ability to rapidly screen for variants of entomopathogenic fungi with enhanced desirable traits, e.g., increased stress tolerance and increased virulence, has the potential for generating isolates via non-genetic modification (non-GMO) approaches for increasing the utility and application of insect-pathogenic fungi for biological pest control. Screening of UV-induced mutants for greater UV and thermal tolerances has been used on a number of occasions for enhancing entomopathogenic fungi (10, 19, 20). In addition, several studies have examined the consequences of UV exposure for M. anisopliae virulence (15, 16, 21). These have indicated that for some entomopathogenic fungi, variations in tolerances to UV-B radiation and heat are potentially linked to the levels of conidial pigmentation (22, 23). Along these lines, from a screen of >6,000 colonies derived from exposure of wild-type M. anisopliae to UV radiation for 40 min, a number of mutants were isolated that showed more UV tolerance, rapid colony growth, and increased virulence (14). Analyses of cell-free culture supernatants from one of these mutants (MaUV-HV) indicated changes in secondary metabolite production. Critically, the mutant also displayed increased virulence, and the toxicity and insecticidal activity of ethyl acetate extracts from MaUV-HV cell-free supernatants were ~20 times greater than those of extracts derived from the wild-type parent (14).

Here, we sought to expand our understanding of the changes that occurred in the MaUV-HV strain that could account for its phenotype by using a proteomics approach that was then coupled to genetic validation of identified protein target changes. Based on analyses of the DAP data set between the M. anisopliae UV mutant strain and the parent wild type, a total of 1,459 proteins with different expression levels, including 693 more abundant and 766 less abundant proteins, were seen in the MaUV-HV strain compared to the wild type. Consistent with the general increased growth (rate) phenotype of the MaUV-HV strain, DAPs belonging to generalized growth and biosynthetic pathways, e.g., ribosome functioning, carbon/nitrogen metabolism, and cell cycle, were found to be more abundant in the mutant. Examples of specific DAPs known to be involved in growth included glutamine synthetase, condensin complex component Cnd2, and a Mob1/phocein family protein (24, 25). Less abundant proteins in terms of general pathways appeared to be more enriched in glycolysis, gluconeogenesis, cysteine and methionine metabolism, and glutathione metabolism, indicating potential shifts in energy mobilization and nutrient assimilation pathways that favor faster growth. Consistent with the increased stress resistance phenotype seen for the MaUV-HV isolate, the more abundant DAP data set included heat shock and oxidative stress-mediating proteins, cyclophilins, and a variety of metabolism-related proteins, all of which represent factors involved in mediating responses to general stress, oxidative and osmotic stress, and UV irradiation stress (25–28). In several instances, different family members of stress response genes were differentially abundant; thus, several GST genes were more abundant in the MaUV-HV strain, whereas others were less abundant. These data imply that there may be trade-offs in the expression of some stress response genes in the mutant strain. With respect to virulence, several critical factors were found to be differentially abundant. These included members of the LysM family (29, 30), glycosyl hydrolases, implicated in cuticle degradation and host carbon utilizations (30), and the cytochrome P450 family and acyl-CoA dehydrogenase, implicated in the synthesis of insect toxins (e.g., destruxin) and host fatty acid metabolism (31). Furthermore, elevated levels of glycosyl hydrolases and the cytochrome P450 CYP5058A4 pathogenicity factors were seen for the MaUV-HV strain. However, a number of pathogenicity factors were less abundant in terms of protein levels in the mutant. These included an isopropylmalate dehydrogenase, several cytochrome P450 enzymes, enzymes involved in fatty acid metabolism processes (31, 32), an FPP synthase (annotated as *Ma*FPPS1), and a GGPPS synthase (annotated as *Ma*GPPS5), the latter involved in terpenoid metabolite processes (33, 34).

Terpenoid biosynthetic pathways link the production of essential sterols (ergosterol) to components of membranes, different biological functions, and various secondary metabolites (33, 35). As these processes act as a critical hub for many downstream products, we sought to examine the nucleotide sequences of select less abundant genes, particularly because of the nature of how the MaUV-HV strain was isolated, i.e., by a mutagenic process that would result in genome-wide changes. We identified a total of 11 differentially expressed proteins (out of 20 proteins examined in the terpenoid backbone biosynthesis pathway). Of these, six showed a range of single nucleotide changes (mutations) that affected the ORF and subsequent protein sequences. In particular, changes in farnesyl pyrophosphate synthase (*MaFPPS1*) and geranylgeranyl diphosphate synthase (*MaGGPPS*5) genes were noted. Both of these enzymes are involved in isoprenoid biosynthesis via classical mevalonate (MVA) pathways (36) and contribute to protein farnesylation/geranylgeranylation, which are required for the downstream activation of a range of small GTPases, including members of the Rab, Ras, and Rho/Rac families. In addition, they produce the products that serve as precursors to sterols, sesquiterpenes, dolichols, carotenoids, diterpenes, some mitochondrial ubiquinones, plant chlorophylls, geranylgeranylated proteins, and even archaeal ether-linked lipids (32). FPP synthase is a branch-point enzyme that can direct carbon flow away from the central portions of isoprenoid pathways (37). We have recently characterized the *MaGGPPS5* gene, and the Δ*MaGGPPS5* mutant strain showed faster growth and greater virulence than the wild type (18). This strain was almost as good as the MaUV-HV in terms of growth, conidiation, and stress response but increased in virulence. As our data identified *MaFPPS1* as another potential mutation that contributes to the MaUV-HV phenotype, we constructed a targeted gene knockout of *MaFPPS1*. This mutant was strikingly similar (but with important differences) to the Δ*MaGGPPS5* strain, namely, in that it showed faster conidial germination, faster growth, and increased virulence compared to the wild type and was also almost as good as MaUV-HV and even increased in virulence. The levels of the destruxins, important secondary metabolites known to display a range of toxicity toward various insects, increased by 15- to 50-fold in the Δ*MaFPPS1* mutant. The Δ*MaFPPS1* mutant also accumulated subglutinol C, which was essentially not found in the wild type, as well showing as an ~100-fold increase in an as-yet-uncharacterized compound, but it lost production of subglutinol analog 45 compared to the level in the wild-type parent strain. However, the levels of these compounds were similar to the levels detected in MaUV-HV. In yeast, FPPS produces both farnesyl pyrophosphate (FPP) and geranyl pyrophosphate, with FPP being a feedback regulator of mevalonate synthesis targeting degradation of 3-hydrox-3-methylglutary-CoA reductase (HMGR). Fluctuations in FPP levels are thought to have greater effects on polyprenol synthesis than sterol levels (38), and the mevalonate pathway acts as the route of synthesis for a wide range of terpenoids, e.g., mycotoxins, trichodermin, and harzianum, that participate in the biocontrol activity of certain Trichoderma spp. (39). Due to the presence of multiple *FPPS* genes in Metarhizium spp., it is difficult to indicate exactly why the Δ*MaFPPS1* mutant appears to redirect secondary metabolite synthesis to destruxins and subglutinols (and potentially other compounds); however, the accumulation of (at least some of) these compounds is consistent with and helps provide a mechanistic explanation for (some aspects of) the MaUV-HV phenotype. Our data suggest that the combined mutation of *MaGGPPS5* and *MaFPPS1* may be the most (or at the very least highly) significant contributors to the overall MaUV-HV phenotype.

The method used for isolating the strain of the insect-pathogenic fungus, M. anisopliae, used in this study, as well as similar methods used by others, relied upon a simple mutagenesis protocol and screening for faster growth (14). However, the underlying mechanism(s) and/or changes that occurred in the mutant (or mutants of this nature) have largely remained a “black box,” with few studies attempting to account for the phenotypes observed. Here, we have used a proteomics approach to uncover cellular processes that can illuminate the nature of this black box. Our data provide unique insights into proteomic changes that can help account for the phenotype of the hypervirulent M. anisopliae strain isolated. As using proteomics is, in this case, essentially nontargeted, a less biased outcome in terms of data analyses, i.e., changes seen in the mutant compared to the wild type, can be obtained. This led to the identification of two genes previously unknown to be involved in mediating three important physiological processes, namely, fungal stress, growth, and virulence. The contributions of one, *MaGPPS5*, have recently been genetically verified, and here we expand this genetic characterization to the *MaFPPS1* gene. We show that specific changes in the proteome could help account for specific phenotypes seen in the mutant, including faster growth, increased UV and heat tolerances, and increased virulence, and confirm the contribution of the gene via genetic analyses. Based upon these proteomics data, specific pathways not previously known to impact virulence can be further examined. These include, in particular, genes involved in terpenoid biosynthesis, glutathione and nitrogen metabolism, and fatty acid utilization.

## MATERIALS AND METHODS

### Preparation of fungal protein extracts.

Conidia of the M. anisopliae wild-type strain (SM04, CCTCC accession number M2016250) and MaUV-HV were prepared as described previously (14). For routine growth, fungi were cultivated in Czapek-Dox broth/agar (CZB/CZA), potato dextrose broth/agar (PDB/PDA), Sabouraud dextrose agar (SDA), and Sabouraud dextrose agar with 0.5% yeast extract (SDAY), as indicated. Fungal conidia (5 mL, 1 × 10^7^ conidia mL^−1^) from the wild-type strain (SM04 [Ma-WT]) and UV mutant (MaUV-HV) were inoculated into 1-L flasks containing 300 mL of Czapek-Dox broth (CZB) supplemented with 0.5% peptone (CZP), and incubated, with aeration (180 rpm), at different temperatures (23, 25, 27, 29, and 31 ± 1°C) for 3 days for the production of the seed inocula. The seed inocula were harvested by filtration (0.45-μm Millipore filter), rinsed 3 times with sterile ddH_2_O to removed residual medium, and then transferred to Czapek-Dox broth (900 mL) for another 7 days of incubation. At the indicated time points (3, 4, 5, 6, and 7 days), aliquots (50 mL) of the growing cultures were taken and fungal cells (fungal mycelium) were harvested by centrifugation (12,000 × *g* for 15 min) and stored at −20°C until use. The cell-free culture supernatant was also kept, filtered (0.45-μm Millipore filter), and stored at 4°C until use.

The total protein contents from the cell-free culture supernatants and the mycelial pellets were extracted using trichloroacetic acid (TCA) and acetone as described previously (14). Briefly, three volumes (10 mL) of precooled 15% (wt/vol) TCA-acetone was added to the filtered culture supernatant or directly added to the harvested mycelia in a crucible, and for the latter, ground into a powder. After mixing, the expected proteins were collected by centrifugation (3,000 × *g* for 10 min at 4°C). Protein pellets deposited in the bottom were washed 3 times with 1 mL prechilled acetone, and extracts were stored at 4°C until use. Protein extracts were analyzed by sodium dodecyl sulfate polyacrylamide gel electrophoresis (SDS-PAGE), and gels were generally stained using the Coomassie brilliant blue method as described previously (40). Protein concentrations were measured using the Bradford Coomassie brilliant blue G-250 method with BSA as the standard (41). All experiments were performed in triplicate, and experiments were repeated with at least one independent batch of conidia as the inoculum. Data are presented as mean values ± standard errors (SE). Proteins derived from the culture conditions (25 ± 1°C for 5 days) were used for further experiments as described.

### Protein sample digestion and TMT labeling.

For comparison of protein expression differences between the MaUV-HV and wild-type strains, protein digestions were performed using the filter-aided sample preparation (FASP) protocol as described previously (42). The resulting peptide mixtures were labeled using the tandem mass tag (TMT) reagent following the manufacturer’s recommendations (Applied Biosystems), and all samples were analyzed at Shanghai Applied Protein Technology. Briefly, 200 μg of protein/sample was mixed with 30 μL STD buffer (4% [wt/vol] SDS, 100 mM dithiothreitol [DTT], and 100 mM Tris-HCl, pH 7.6). Detergent, DTT, and other low-molecular-weight components were removed by repeated ultrafiltration (Microcon centrifugal filter units, 30 kD) using UA buffer (8 M urea, 150 mM Tris-HCl, pH 8.0). Samples were then mixed with 100 μL 0.05 M iodoacetamide in UA buffer to block reduction of cysteine residues. Samples were then incubated in the dark for 20 min. Sample filters were washed 3 times with 100 μL UA buffer, followed by 2 washes using 100 μL DS buffer (50 mM triethylammonium bicarbonate at pH 8.5). Protein suspensions (40 μL in DS buffer) were then digested with trypsin (2 μg; Promega) overnight at 37°C, and the resulting peptides were collected as a filtrate. Total peptide content was evaluated by UV_280 nm_ measurement using an extinction coefficient of 1.1 of 0.1% (g/L) calculated on tryptophan and tyrosine frequency in vertebrate proteins. For labeling, TMT-sample reaction mixtures were dissolved in 70 μL of ethanol and added to the respective peptide mixture. The samples (100 μg per treatment) were labeled with TMT-126, -127, and -128 and TMT-129, -130, and -131 for the protein groups from the wild-type and MaUV-HV strains from three biological replicates and were vacuum dried.

### Peptide fractionation with SCX chromatography.

TMT-labeled peptides were fractionated by strong cation exchange (SCX) chromatography (ÄKTA purifier system; GE Healthcare), using the protocol in detail as described previously for all samples (43). All samples were stored at −80°C until further analyzed by liquid chromatography-mass spectrometry (LC-MS).

### LC-electrospray ionization (ESI)-MS/MS analysis by Q Exactive.

Experiments were performed on a Q Exactive mass spectrometer that was coupled to an Easy nLC (Proxeon Biosystems, now Thermo Fisher Scientific). The column was equilibrated with 95% buffer A (0.1% formic acid). Ten microliters of each fraction was injected for nanoscale LC (nanoLC)-tandem mass spectrometry (MS/MS) analysis. The peptide mixture (5 μg) was loaded onto a Thermo Scientific Acclaim PepMap 100 reverse trap phase column (100 μm by 2 cm nanoViper C_18_) and separated with a linear gradient of buffer B (84% acetonitrile and 0.1% formic acid) at a flow rate of 300 nL/min by using an analytical column (Thermo Scientific EASY-Column, 10 cm long, inner diameter 75 μm, 3-μm resin, C_18_-A2) over 90 min. The mass spectrometer was operated in positive ion mode. MS data was acquired using a data-dependent top-10 method dynamically choosing the most abundant precursor ions from the survey scan (300 to 1,800 *m/z*) for high-energy collisional dissociation (HCD) fragmentation. Determination of the target value was based on predictive automatic gain control (pAGC); the AGC target was set to 1e6, and the maximum injection time to 50 ms. The dynamic exclusion duration was 60 s. Survey scans were acquired at a resolution of 70,000 at *m/z* 200, and the resolution for HCD spectra was set to 17,500 at *m/z* 200 and the isolation width to 2 *m/z*. The normalized collision energy was 30 eV, and the underfill ratio, which specifies the minimum percentage of the target value likely to be reached at maximum fill time, was defined as 0.1%. The instrument was run with peptide recognition mode enabled.

### Sequence database searching and data analysis.

NanoLC-MS/MS spectra were searched using MASCOT engine version 2.2 (Matrix Science, London, UK) embedded into Proteome Discoverer 1.4 (Thermo Electron, San Jose, CA) against UniProt Metarhizium anisopliae 22617 20180809.fasta, and all samples were analyzed at Shanghai Applied Protein Technology. For protein identification, the following options were used. Enzyme = trypsin, missed cleavage = 2, fixed modification: carbamidomethyl (C), TMT 6 plex (N-term), TMT 6 plex (K), variable modification: oxidation (M), TMT 6 plex (Y), peptide mass tolerance of ±20 ppm, LC-MS/MS tolerance of 0.1 Da, decoy as the database pattern, peptide false discovery rate of ≤0.01, and each identified protein having at least one unique peptide. The protein ratios were calculated as the median of only the unique protein peptides. Experimental bias was performed to normalize all peptide ratios based on the median protein ratio. The median protein ratio was required to be 1 after normalization.

Student’s *t* test was used to compare the wild-type and MaUV-HV mutant groups. In addition, only those proteins with *P* values of <0.05 and with >1.40- or <0.71-fold change were classified as differentially abundant proteins (DAPs). The DAPs were mapped to the Kyoto Encyclopedia of Genes and Genomes (KEGG) pathways (http://www.genome.jp/kegg/) and to Gene Ontology (GO) terms (http://www.geneontology.org). The protein-protein interaction networks involving the DAP data set were retrieved using the IntAct molecular interaction database (http://www.ebi.ac.uk/intact/) by their STRING software (http://string-db.org/). Graphical visualizations and interaction network analyses were performed in Cytoscape (http://www.cytoscape.org/, version 3.2.1). KEGG and GO pathway enrichment analyses were examined using Fisher’s exact test using the entire quantified protein annotations as the background data set. Derived *P* values were adjusted using the Benjamini-Hochberg correction applied for multiple testing. Only categories and pathways with *P* values of <0.05 were considered statistically significant.

### Identification of gene mutations in the MaUV-HV strain.

Mutations in the nucleotide sequences of genes encoding 11 differentially abundant proteins involved in terpenoid backbone biosynthesis were examined (Table 1). DNA and amino acid sequences of the wild-type target proteins were downloaded from UniProt Metarhizium anisopliae 22617 20180809 and NCBI according to the protein identification codes and used to design primers for the amplification of target genes from the wild-type and MaUV-HV strains (Table S3). Briefly, M. anisopliae genomic DNA and total RNA were isolated from growing mycelia of the wild-type and MaUV-HV strains. Fungal cultures were inoculated in CZP and grown in flasks (4 to 5 days at 200 rpm and 26 ± 1°C) before harvesting and DNA or RNA extraction. Target genomic DNA sequences (gDNA, i.e., open reading frame plus any introns) was amplified by PCR using genomic DNA as the template and subsequent sequencing of the cloned fragments (Life Technologies, Shanghai, China). Total RNA was isolated from mycelium samples using the TRIzol reagent (Invitrogen, Shanghai, China). Total cDNA was synthesized according to the manufacturer’s instructions (SMART [switching mechanism at 5′ end of RNA transcript] rapid amplification of cDNA ends [RACE] cDNA amplification kit; Clontech). The full length of the cDNA sequence of the target gene was amplified with the cDNA-specific primers (3′-RACE cDNA amplification primers, Table S5) and the oligo(dT)12-18 primer supplied by the SMART RACE cDNA amplification kit. Cloned fragments were sequenced as described above.

### Nucleic acid manipulations.

The primer pairs used for nucleic acid manipulations are listed in Table S4. An internal fragment (2,172 bp) of the farnesyl pyrophosphate synthetase (FPPS-M) (M. anisopliae tr|A0A0B4FZV1| MAN_08993 gene sequence) was obtained by amplification using the genomic DNA from M. anisopliae wild type and primers FPPS1-F/R. The full-length *MaFPPS1* gene sequence was subsequently amplified using the primer pairs FPPS1-LF/RR. The sequence integrity of all PCR products was confirmed by DNA sequencing integrity and the resultant data used to assemble the complete genomic sequence of *MaFPPS1* gDNA.

The M. anisopliae targeted gene knockout mutant was constructed by cloning of 5′ and 3′ flanking sequences of the *MaFPPS1* gene by PCR using template genomic DNA from M. anisopliae wild type with primer pairs FPPS1-LF/LR (1.59 kb) and FPPS1-RF/RR (2.02 kb) to obtain 5′ and 3′ gene flanking sequences, respectively. Plasmid pHS-*Bar*-PX was used to construct the vector for homologous recombination using the enzyme sites HindIII/SpeI at the left side of the *PtrpC* gene and the PstI/XhoI sites at the right side of the *TtrpC* gene (Fig. S5A). Flanking sequences (5′ and 3′) of the *FPPS1* gene were separately subcloned into the restriction enzyme sites HindIII/SpeI and PstI/XhoI in plasmid pHS-*Bar*-PX, yielding plasmid pFPPS1 carrying the 5′ sequence + *PtrpC* gene + *Bar* gene + *TtrpC* gene + FPPS1 3′ sequence. The vector construct was transformed into host cells (A. tumefaciens strain LBA4404), and the resultant strain used to transform wild-type M. anisopliae, using selection for phosphinothricin (200 μg/mL) resistance (*bar* gene marker) as described below.

For complementation, the construct p*Sur-FPPS1* was assembled using the entire *FPPS1* gene along with promoter sequences (4.24 kb total) by PCR amplification using primers FPPS1-CF/-CR and a template from M. anisopliae gDNA. The product of PCR was ligated, using In-Fusion DNA ligase, into the restriction enzymes sites XbaI and PstI in plasmid pK-*Sur-GFP* (Fig. S5B). The resultant construct, p*Sur-FPPS1*, was then transformed via AMT into the Δ*MaFPPS1* mutant to make the complemented strain, Δ*MaFPPS1*::*FPPS1*.

A strain constitutively expressing the *FPPS1* gene was constructed as follows: the *FPPS1* gene (1,032-bp ORF sequence) was amplified by PCR using primers FPPS1-kvF/-kvR and template from M. anisopliae cDNA and cloned into the restriction enzymes sites (SpeI and XbaI) in plasmid pT-*Sur-GFP* to yield p*Sur-FPPS1-orf* (replacing the *GFP* gene) (Fig. S5C). Plasmid p*Sur-FPPS1-orf* was transformed into the M. anisopliae wild-type strain using the Agrobacterium-mediated transformation (AMT) method, yielding the *FPPS1*^Const^ strain. All transformants were single-spore purified, and the expected integration events verified by PCR and Southern blotting.

### AMT.

M. anisopliae was transformed using the Agrobacterium-mediated transformation (AMT) method (A. tumefaciens strain LBA4404) as described previously (40, 44), with putative transformants initially isolated for the expected integration event by PCR using appropriate primer pairs (e.g., FPPS1-kvF/-kvR for the gene knockout construct). Clones showing the expected PCR fragment sizes in the Δ*FPPS1* mutant (e.g., 2.02 kb = 1.44-kb sequence of the *bar* gene cassette inserted into 0.59 kb [1.21 − 0.62 = 0.59] of the *FPPS1* gene) were selected and sequenced to confirm the desired integration events. The desired integration events were confirmed by PCR and Southern blotting. Southern blots were performed using the digoxigenin (DIG)-high prime DNA labeling and detection starter kit II in conjunction with chemiluminescent detection (Roche, Penzberg, Germany). Primers for initial colony verification and for use in probe amplification are listed in Table S6. Real-time quantitative PCR was performed as follow: total RNA was extracted from the mycelium of the three different strains (wild-type, Δ*FPPS1*, and *FPPS1*^Const^ strains) cultured in CZB supplemented with insect nymph extract (1%, S. furcifera) and grown for 4 to 7 days at 26 ± 1°C, using the total RNA extraction kit (Omega) following the manufacturer’s instruction. Insect extracts were prepared as follows: 1% (wt/wt) of S. furcifera was ground on ice and then put in CZB and sterilized at 121°C for 20 min. RT-quantitative PCR (qRT-PCR) was performed using Champagne *Taq* DNA polymerase in a LightCycler 480 (Roche, Indianapolis, IN, USA). All experiments were performed with three replications, and the relative transcript levels of target genes were normalized to the level of *β*-*actin* (GenBank accession no. MN106223.1) and calculated using the cycle threshold (2^−ΔΔ^*^CT^*) method (45).

### Fungal growth and phenotypic assays.

Fungal growth was measured by spotting fungal spore suspensions (2 to 5 μL of 1 × 10^6^ conidia/mL in 0.05% Tween 80) on solid media, i.e., SDAY, PDA, SDA, and CZA. Plates were incubated for 7 to 12 days at 26 ± 1°C, and colony size and morphology examined/measured every day. Spore germination was examined microscopically by counting the number of germinated spores out of the number of total spores, where germination was determined to have occurred when the germ tube length was equal to or greater than the spore diameter/total number of spores under the conditions tested. All experiments were performed with three technical replicates, and the entire experiment was repeated three times using different conidial batches as the inoculum. Data are presented as mean values ± SE. Fungal tolerances to UV-B irradiation and heat shock (HS) were determined as described previously (2). The length of time for 50% of the conidia to have germination (GT_50_) values was calculated for indicated conditions for each strain.

### Production of fungal secondary metabolite extracts.

Conidia from M. anisopliae strains Ma-WT, MaUV-HV, and the Δ*MaFPPS1* mutant were inoculated (5 mL, 1 × 10^7^ conidia mL^−1^) into culture flasks (400 mL in 1-L flasks) containing CZP (CZB plus 0.5% peptone). Fungal cells and mycelia were removed by centrifugation (12,000 × *g* for 15 min), and the resultant fungal-cell-free culture supernatant was filtered through a 0.45-μm Millipore membrane and stored at 4°C until use.

Extraction of fungal metabolites was performed using ethyl acetate (EthOAc) as described previously, and extracts were stored at −20°C until use (14). The metabolite analyses and the EthOAc extractions were performed using the method in detail as described previously (18). Subglutinols A, B, C, and analog 45 were identified as described previously (46, 47). Standards for destruxins and other molecules were used as indicated (Sigma-Aldrich Company Ltd.).

### Insect bioassays.

The lethal times to kill 50% (LT_50_) and the mean lethal concentrations to kill 50% (LC_50_) of treated insects were determined for indicated fungal strains as described previously using several different hosts (40). For the insect S. furcifera, LT_50_ values were determined using adults and 4th instar nymphs immersed for 10 s in a fungal concentration of 1 × 10^7^ conidia/mL (topical bioassay), and LC_50_ values were determined using adults and 4th instar nymphs immersed for 10 s in different fungal conidial concentrations (1 × 10^3^ to 1 × 10^8^ conidia/mL). Control and treated insects were placed in standard petri dishes containing rice seedlings, and the plates were incubated at 26 ± 1°C in bioassay chambers under 75% to 90% relative humidity and a 14 h/10 h (light/dark) photoperiod. Three replicates of 30 S. furcifera insects (nymphs or adults) were used for each treatment. Mortality was recorded every 24 h until any adult emergence from surviving nymphs or up to 12 days posttreatment for bioassays using adults. Any dead insects were immediately removed from the bioassay chambers and placed on premoistened clear paper in Petri dishes to confirm fungal development and sporulation on cadavers. Fungal conidial production on the host cadaver was quantified as described previously (48). Experiments were repeated three times using fresh conidial suspensions and different batches of insects. Insect bioassays were also performed, using Pieris rapae 4th instar larvae as the host, by (i) topical infection (as described above) or (ii) by intrahemocoel injection. For topical bioassays, P. rapae larvae were immersed for 10 s in conidial suspensions adjusted to 1 × 10^7^ conidia/mL in 0.05% Tween 80–sterile distilled water (dH_2_O). For intrahemocoel injection assays, 10-μL amounts of conidial suspension at the same concentration were injected via abdominal segments into the host hemocoel. Each experiment consisted of three technical replicates of 30 insects/treatment group, and the whole experiment was repeated three times.

The insect toxicity of fungal extracts (ethyl acetate) was tested by injection (intrahemocoel via the abdomen) into Pieris rapae 4th instar larvae. Ten-microliter aliquots of samples adjusted to serial concentrations of 0.125, 0.25, 0.5, 1, and 5 ppm of each respective fungal extract were dissolved in double-distilled water (ddH_2_O) prior to injection. Controls were treated (injected) with ddH_2_O or 0.05% Tween 80–ddH_2_O. Morbidity was recorded at 12-h intervals posttreatment. Each treatment group consisted of 30 to 35 insects with 3 treatment groups per test condition, and the entire experiment was repeated 3 times.

### Data analyses.

GenBank NCBI BLAST, the DNAMAN software package (version 6.0; Lynnon BioSoft, Canada), MEGA version 6.0 (http://www.megasoftware.net), and ProtParam (http://us.expasy.org/tools/protparam.html), were used for bioinformatic and phylogenetic analyses. FPPS1 amino acid sequences from different fungal species were obtained from NCBI. Insect mortality data were corrected using Abbotts’ formula (1925), and curves of log concentration minus Probit line (LC-p) and log time minus Probit line (LT-p) were calculated and tested using the chi-square test. Median lethal concentration (LC_50_) and median lethal time (LT_50_) values and their confidence intervals were determined using Probit analysis using SPSS 8.0 for Windows. Quantifications of spore germination, mycelial growth, and conidial production were compared by one-way analysis of variance (ANOVA) and mean values using Tukey’s Student range test (Tukey’s, *P = *0.05) (SAS Institute, Inc., Cary, NC, USA).

### Data availability.

The mass spectrometry proteomics data have been deposited to the ProteomeXchange Consortium (http://proteomecentral.proteomexchange.org) via the iProX partner repository with the data set identifier PXD033657. Our data can be found online at https://www.iprox.cn/page/project.html?id=IPX0004355000.
