# Supplementary material for: Proteomic Analysis of a Hypervirulent Mutant of the Insect-Pathogenic Fungus Metarhizium anisopliae Reveals Changes in Pathogenicity and Terpenoid Pathways
Source: Microbiol Spectr. 2022 Oct 31;10(6):e00760-22. doi: 10.1128/spectrum.00760-22 (PMC9769655; doi:10.1128/spectrum.00760-22)
Supplement: Supplemental file 1 — Fig. S1 to Fig S5; Tables S1 to S4. Download spectrum.00760-22-s0001.pdf, PDF file, 0.5 MB [file spectrum.00760-22-s0001.pdf]

Supplemental Material FOR publication

Supplemental Figures

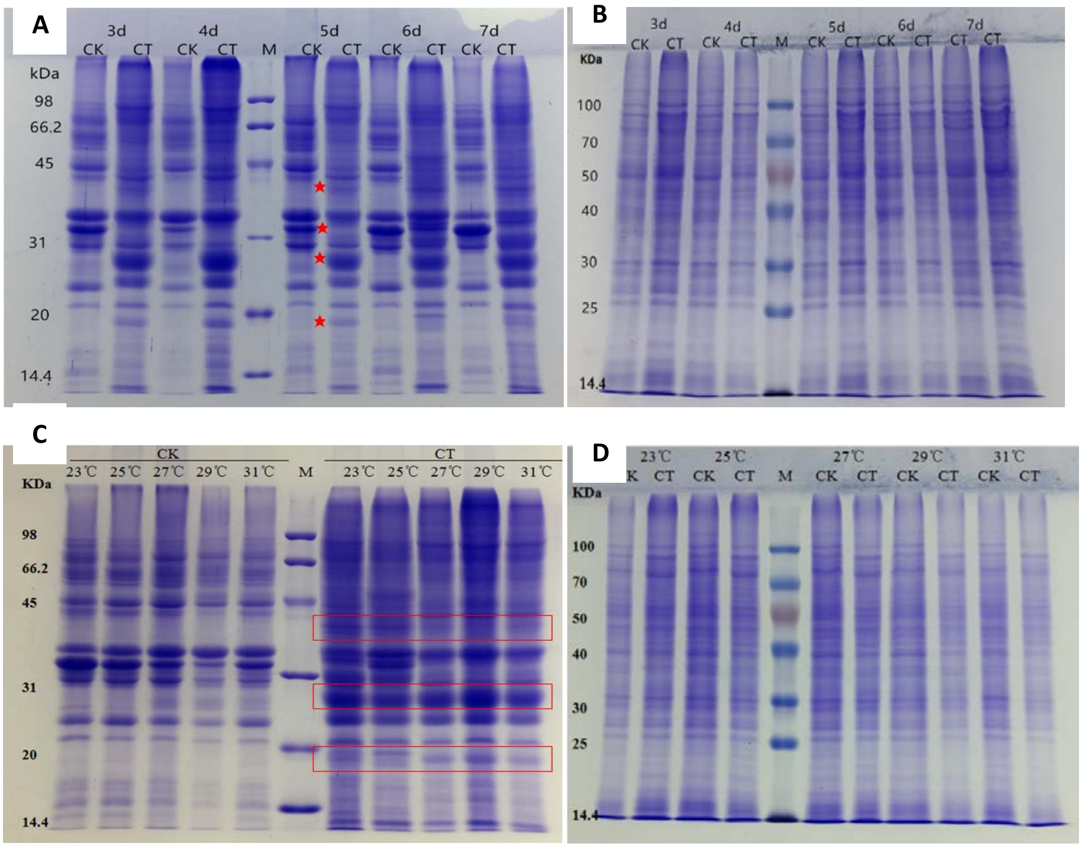

Supplemental Fig. S1. A. Secreted protein resulted from *M. anisopliae* wild-type strain (CK) and MaUV-HV mutant (CT). **B.** Mycelial protein resulted from *M. anisopliae* wild-type strain (CK) and MaUV-HV mutant (CT). **C.** Secreted protein resulted from *M. anisopliae* wild-type strain (CK) and MaUV-HV mutant (CT). **D.** Mycelial protein resulted from of *M. anisopliae* wild-type strain (CK) and MaUV-HV mutant (CT). SDS-PAGE of protein derived from mycelium and filtered after 5 days at 23, 25, 27, 29, 31°C of culture. M = molecular weight standards.

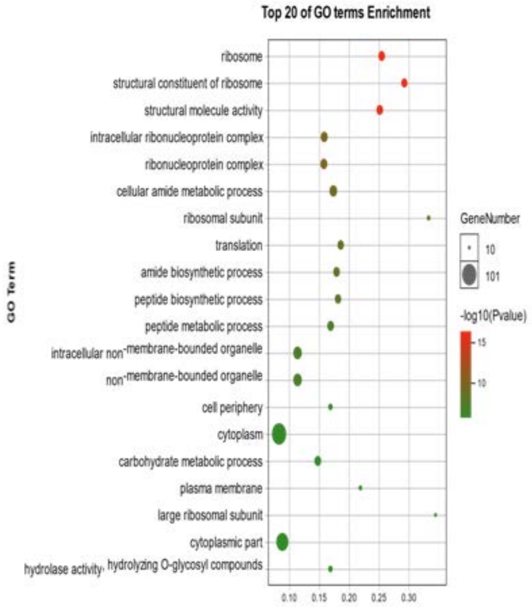

Up regulated proteins enrichment of level 3 Go terms

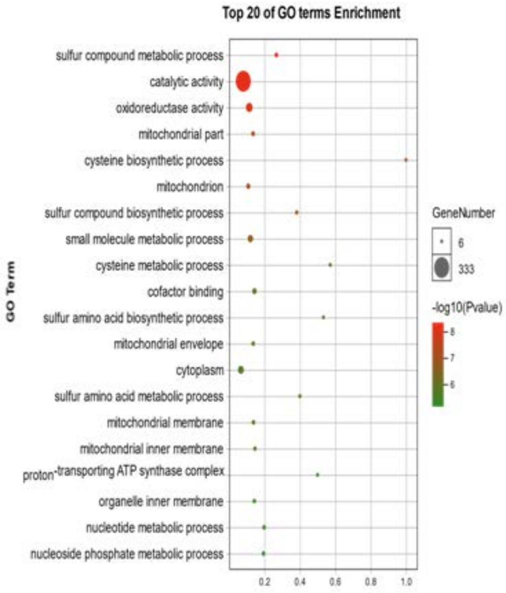

Down regulated proteins enrichment of level 3 Go terms

Supplemental Fig. S2. GO analysis showed with up/down abundant proteins in level 3 Go term Notes: Level 3 GO term enrichment was shown above.

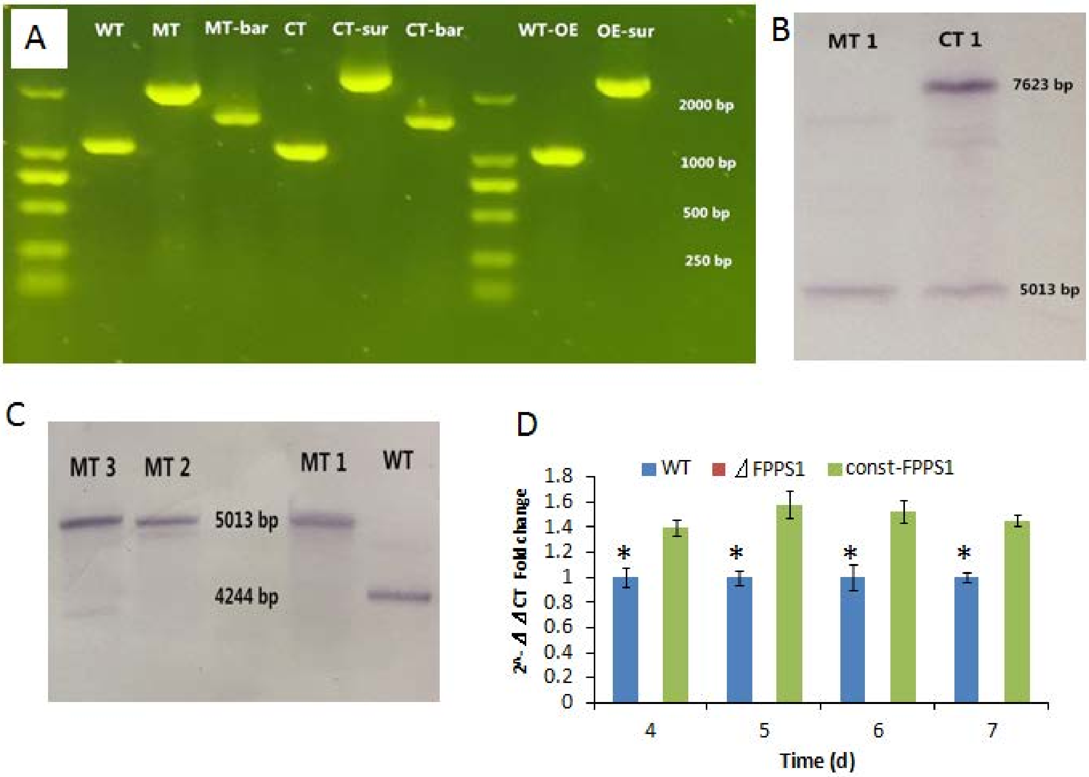

Supplemental Fig. S3. Southern blot, qRT-PCR and PCR identification of the expected correct integration events. **A.** PCR identification of the expected correct integration events in the Δ*FPPS1*, complemented and *FPPS1*^{const} strains. Lanes in figure are labeled as follows; WT (wild type, 1.2kb), MT (*ΔFPPS1*, 2.02kb), CT (*ΔFPPS1::FPPS1*, 1.2kb), MT/CT-Pbar (1.4kb), WT-OE (1.2kb), OE-sur (2.53 kb). PCR products amplified using primers FPPS1-kvF/-kvR and genomic DNA from the indicated strains, desired sizes for wild type parental strain and ectopic complemented strain are 1.2kb, and for the correct knockout mutant integration events is 2.02 kb (1.44 kb, *bar* gene cassette sequence inserted into *ΔFPPS1*, 1.21-0.62=0.59 kb), and for the ectopic complemented integration events is 2.53 kb (2.53 kb sequence of *sur* gene); *FPPS1*gene constitutive expression in parental wild type (WT-OE, 1.2kb), *sur* gene in *MaFPPS1*^{const} strain (OE-sur) with 2.53 kb of *sur* gene sequence. **B. & C.**

Southern blotting verification of constructed strains. Genomic DNA from different fungal strains (WT, MT, CT) was digested by *Hind* III / *Xho* I, and fragments were isolated on an 0.8 % agarose gel and transferred to a Biodyne B nylon membrane. A 1.2 kb fragment related to the *FPPS1* gene was derived from *M. anisopliae* gDNA and used as probe for verification WT and MT1-3 strains, and a 1.4 kb fragment related to the *bar* gene cassette was used as probe for verification of the MT1 and CT1 strains. Target fragments were as follows; in WT 4.2kb (including 5' flank + ORF + 3'-flank), deletion mutant (MT1-3) fragment 5.0 kb (including 5' flank + *bar* gene cassette + 3'-flank), ectopic complement mutant (CT) fragment 7.6 kb (including *bar* and *sur* gene cassette + ORF + 5' flank + 3'-flank). **D.** Relative gene expression levels of the *FPPS1* gene in the WT, *ΔFPPS1*, and *MaFPPS1* constitutive expression (const-FPPS1) strains grown in insect induction media (CZB + 1.0 % insect nymph) for 4, 5, 6 and 7 days at 26 ^{o}C.

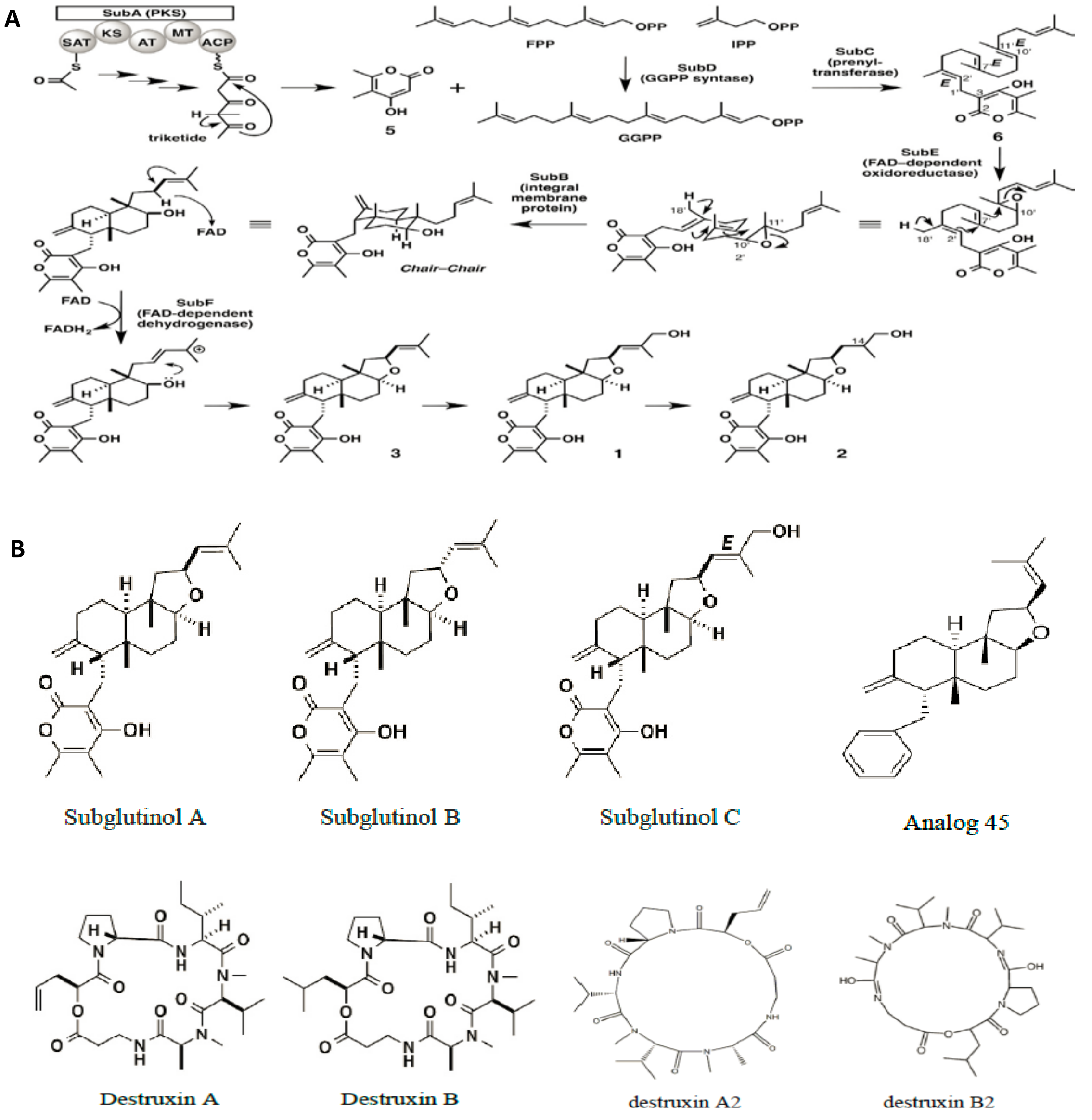

Supplemental Fig. S4. (A) The biosynthetic pathway of subglutinol metabolite (cited from Kato et al., 2016). (B) Structures of subglutinols, analog 45 (product of subglutinol pathway), and a series of fungal destruxins (cited from Kato *et al*., 2016; Park *et al*., 2020).

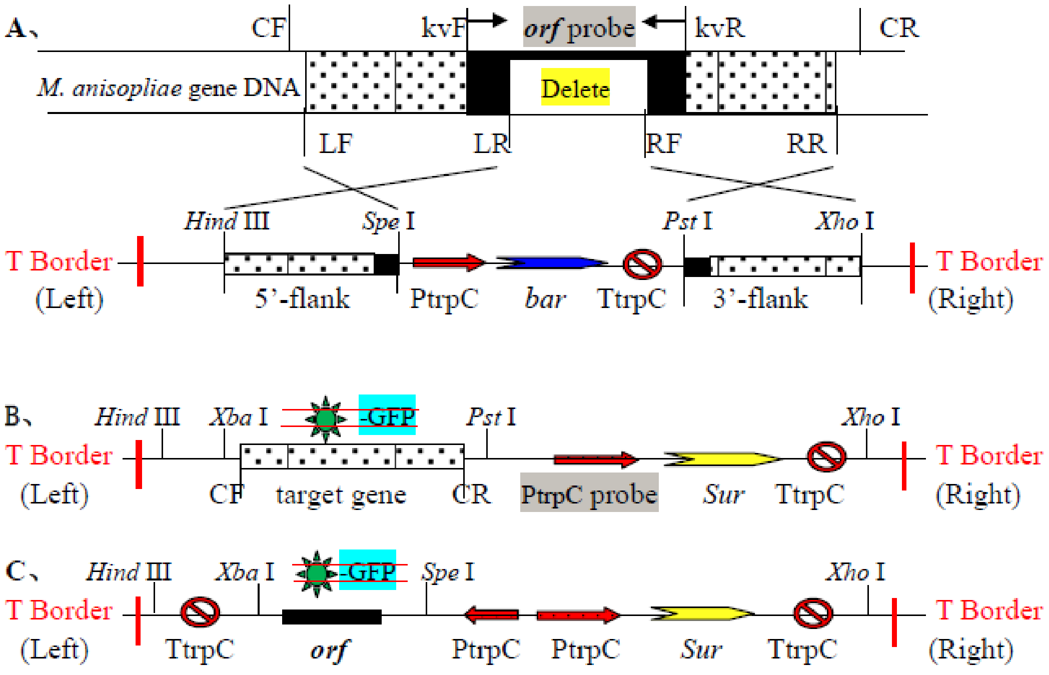

Supplemental Fig. S5 Schematic diagram of the targeted gene *MaFPPS1* reintegrate event. A、Schematic diagram of the construction vector pHS-*bar*-PS, including target gene locus, homologous recombination event, PCR primer sites, ORF probe fragement for the Southern blot analyses. B、Structure diagram of the complementation vector pK-*Sur*-*GFP*, showing the *sur* and target gene (*GFP* gene replacement) locus and homologous recombination event. The PtrpC probe used for the Southern blot analyses is shown. C、Schematic diagram of the constitutive expression vector, pT-*Sur*-*GFP*, showing the *sur* and target gene (*GFP* gene replacement) locus event. The sequences of the primers designed to the LF / LR, RF / RR, kvF / kvR, and CF / CR sequences are listed in Supplemental Table S4.

Supplemental tables S1-4

Supplemental Table S1. Fungal proteins derived from *M. anisopliae* wild-type and MaUV-HV mutant strains cultured in different time（mg/mL）(27±1 ℃，CZB)

Time	Protein from cell free supernatant		Protein from fungal mycelium	
	Ma-WT	MaUV-HV	Ma-WT	MaUV-HV
3d	0.134±0.007 d	0.183±0.007 d	0.346±0.006 c	0.393±0.004 c
4d	0.165±0.006 c	0.226±0.008 c	0.394±0.005 b	0.421±0.008 b
5d	0.196±0.008 b	0.267±0.010 b	0.412±0.008 ab	0.457±0.007 ab
6d	0.213±0.007ab	0.289±0.008 ab	0.432±0.007 a	0.479±0.010 a
7d	0.236±0.006 a	0.301±0.010 a	0.441±0.006 a	0.480±0.009 a

Means in the same column followed by the same letters are not significantly different (Turkey's HSD test, a = 0.05).

Supplemental Table S2. Fungal proteins derived from *M. anisopliae* wild-type and MaUV-HV mutant cultured in different temperatures（mg/mL）(5d，CZB)

Temp.	Protein from cell free supernatant		Protein from fungal mycelium	
	Ma-WT	MaUV-HV	Ma-WT	MaUV-HV
23 ℃	0.127±0.005 b	0.173±0.003 b	0.271±0.008 b	0.301±0.018 b
25 ℃	0.148±0.004 a	0.206±0.006 a	0.314±0.007 a	0.355±0.014 a
27 ℃	0.142±0.003 b	0.194±0.010 ab	0.302±0.008 ab	0.337±0.019 ab
29 ℃	0.135±0.005 ab	0.174±0.008 b	0.288±0.031 b	0.303±0.047 b
31 ℃	0.128±0.004 b	0.166±0.007 b	0.273±0.007 b	0.288±0.019 b

Means in the same column followed by the same letters are not significantly different (Turkey's HSD test, a = 0.05).

Supplemental Table S3. PCR primers of 9 genes corresponding to the differentially expressed proteins

Protein IDs	DNA Primers / cDNA Primers
A0A0B4FZV1	FZV-F 5’--GTAGTAGTCCGAGGTCAAGAGGGTT--3’<br>FZV-R 5’--ACGAGCCAAATCACTCTCATCACAC--3’<br>cDNA-FZV 5’--AAGATGGATGCCCTGAGACACG--3’
A0A0D9NRB6	NRB-F 5’-- TTTTAGTGCCGGTGGACGTATC--3’<br>NRB- R 5’-- GTCTGTTGTCCGCTGTTTCAGG--3’<br>cDNA-NRB 5’--AATATCCAGCAAACTACACG--3’
A0A0B4GDP2	GDP2-F 5’--GGTATGGATACTCGCTTAGAACTCGG--3’<br>GDP2-R 5’--CGCTCATTCAGCCCTTCAACAA--3’<br>cDNA-GDP2 5’--CAAGCCACCAGACGACCATG--3’
A0A0B4FY87	FY87-F 5’--CACCACGGAAAATGTTCAACGA--3’<br>FY87-R 5’--TCTCAAACTAAAACCCAGCCCC--3’<br>cDNA-FY87 5’-- TGCCATTCTCGACCCTTAACCC--3’
A0A0D9P3F8	P3F8-F 5’-- CCGTGCTTGGATCTTGGTTTGA--3’<br>P3F8-R 5’-- TTTTCTTCCGACCAACTCTTCTACT--3’<br>cDNA-P3F8 5’--GCTTCCCTTCCATCATACCATC--3’
A0A0B4EYR9	EYR9-F 5’--TCTATGACCCAAATGATTTGAACCG--3’<br>EYR9-R 5’--AGCCAAGTCCGTGTCCCCTACC--3’<br>cDNA-EYR9 5’--TGGTCAGCAAGTGTCCACGAT--3’
A0A0B4FNW7	FNW-F 5’-- TCCACCCGTCCGTTGTCTTCAC--3’<br>FNW-R 5’-- ATGTTGGTCGGCCTCCTCTTTG--3’<br>cDNA-FNW 5’-- TCTCCTTTACAACATGGCTTCG--3’
A0A0B4FTF6	FTF6-F 5’--AGGCACAGTTGAAATCGGTTAGAAT--3’<br>FTF6-R 5’--GTTTGCCAATGTTTCCTCAGC--3’<br>cDNA-FTF6 5’--TCGCTTCGCTCTTCCACAAT--3’
A0A0D9NQA4	NQA-F 5’--GAGACGCTGGGAACGGAACAGTGAT--3’<br>NQA-R 5’--GAAGGCGAGGATGCGATAGAAAGGA--3’<br>cDNA-NQA 5’--GGGCTTGCACAAGATGACCAC--3’

Supplemental Table S4. List of primers used in cloning *FPPS1* gene

Primers name	Sequences
FPPS1-F	5'--GTAGTAGTCCGAGGTCAAGAGGGTT---3'
FPPS1-R	5'--ACGAGCCAAATCACTCTCATCACAC---3'
FPPS1-LF	5'-- AACGACGGCCAGTGCCC**AAGCTT**ATGTAACTTCGCCTGTCCTCCCTC--3'
FPPS1-LR	5'--- ATATCAGTTAACGTCG**ACTAGT**GCTTTGATTGTTCCTCGCTCACTTC--3'
FPPS1-RF	5'-ACCAGCCCCTGGGTT**CTGCAG**TCGGGTTGACGAGGTGAAGCAGGTATT--3'
FPPS1-RR	5'--- CATTATTATGGAGAAA**CTCGAG**GCTGCAACGCTGCTACGACCCT---3'
FPPS1-CF	5'-- TTCTACCCAAGCATCGAT**CTGCAG**ATGTAACTTCGCCTGTCCTCCCTC--3'
FPPS1-CR	5'--- AACGTTAAGTGGATCTGC**TCTAGA**GCTGCAACGCTGCTACGACCCT---3'
FPPS1-kvF	5'--ATTCTACCCAAGCATCGAT**ACTAGT**ATGGATGCCCTGAGACACGAGC--3'
FPPS1-kvR	5'--AACGTTAAGTGGATCTGC**TCTAGA**TCGTCTGCAAATCCGTTCCATGTCT--3'
	Verification primers
Psu-F	5'---ATGCTTCGTACTGTTGGCCGC---3'
Bsuy-R	5'---GTCGACGTGAGAGCATGCAATTC---3'
Pbar-F	5'---GACGTTAACTGATATTGAAGGAGC---3'
BarT-R	5'---CCCAGGGGCTGGTGACGGAATTT---3'
SB-F	5'--TCATTGGCAAGACGGGAGGA--3'
SB-R	5'--GCTTGGACGACCTTGTTGATGT--3'
	qRT-PCR primers
Qfpps1-F	5'--- CTACCTGATCCTCGACGACA ---3'
Qfpps1-R	5'--- GAGATCGCACAACTGACCC ---3'
Qactin-F	5'---AGTCCAAGCGTGGTATCCT---3'
Qactin-R	5'---TGGGAGCCTCAGTCAGC---3'

Notes: letters with underline in sequences mean homologous sequence sites. Letters with bold-type in sequences mean enzyme sites
